# The Route to ‘Chemobrain’ - Computational probing of neuronal LTP pathway

**DOI:** 10.1038/s41598-019-45883-9

**Published:** 2019-07-03

**Authors:** Ammad Fahim, Zaira Rehman, Muhammad Faraz Bhatti, Nasar Virk, Amjad Ali, Amir Rashid, Rehan Zafar Paracha

**Affiliations:** 1 Atta ur Rahman School of Applied Biosciences, National University of Sciences and Technology (NUST), Islamabad, Pakistan; 2 Research Centre for Modeling and Simulation, National University of Sciences and Technology (NUST), Islamabad, Pakistan; 3 Department of Biochemistry, National University of Medical Sciences, Rawalpindi, Pakistan; 4 Present Address: EBS Universität für Wirtschaft und Recht, EBS Business School, Rheingaustrasse 1, Oestrich-Winkel, 65375 Germany

**Keywords:** Computational chemistry, Cognitive control

## Abstract

Chemotherapy causes deleterious side effects during the course of cancer management. The toxic effects may be extended to CNS chronically resulting in altered cognitive function like learning and memory. The present study follows a computational assessment of 64 chemotherapeutic drugs for their off-target interactions against the major proteins involved in neuronal long term potentiation pathway. The cancer chemo-drugs were subjected to induced fit docking followed by scoring alignment and drug-targets interaction analysis. The results were further probed by electrostatic potential computation and ligand binding affinity prediction of the top complexes. The study identified novel off-target interactions by Dactinomycin, Temsirolimus, and Everolimus against NMDA, AMPA, PKA and ERK2, while Irinotecan, Bromocriptine and Dasatinib were top interacting drugs for CaMKII. This study presents with basic foundational knowledge regarding potential chemotherapeutic interference in LTP pathway which may modulate neurotransmission and synaptic plasticity in patient receiving these chemotherapies.

## Introduction

Cancer is a multifactorial disease harboring disorders on multiple levels, rendering a single drug to be therapeutically insufficient. Therefore, designing a single drug aiming multiple targets is a difficult proposition. Keeping this in view, multiple targeted combinatorial therapeutic regimens, to achieve enhanced therapeutic efficacy, have been the main stay of cancer treatment. However, employment of drug combinations, may present with increased drug toxicity and varied battery of side effects^1^. Hence, foreseeing the drug side effects and probable drug adverse reactions (ADRs) encountered by patients is difficult by the existing medical literature. This in turn significantly effects quality of life of patients during treatment and disguise the patient well-being even after the treatment is concluded. Such side effects may be because of drug promiscuity or off targets interactions inducing drug-drug or drug-target interactions^2^. As the wealth of information regarding disease state, particularly cancers and its therapies is growing, it is more evident that both on-target and off-target drug-protein interactions need to be taken in account as to predict drug specific side effect profiles. Interestingly, such off targets have been notifying their presence notably in the shape of cardiotoxicity attributable to hERG inhibition^3^ and hepatotoxicity attributably by CYP inhibition^4^.

Therapies for cancer can cause both central and peripheral toxicities leading to a wide differential of cognitive changes which may span from acute onset delirium like symptoms to more progressive degenerative changes and delayed neurological consequences termed as ‘Chemobrain’,‘Chemofog’, ‘Cancer related Cognitive Impairment’ or ‘Chemotherapy induced Cognitive Impairment’ (CICI)^5–10^. Normal human cognitive process constitutes various important neurobiological processes of day to day life like attention, learning, memory, planning and decision making^11,12^. Therefore, any interference may translate into neurodegenerative and psychiatric morbidity. The CICI can exhibit itself in varying forms ranging from but not limited to headache, seizures, acute or chronic encephalopathies, cerebrovascular disease, movement disorders and cranial neuropathies^13^.

Previous studies addressing ‘chemobrain’ can be broadly segregated in to clinical and preclinical experiments^14,15^. The clinical studies engross clinical assessment of cognitive function during and/or after chemotherapy via use of battery of neuropsychological test. Whilst, preclinical histological or behavioral studies investigate any potential connection between chemotherapy associated neurotoxicity and hippocampal neurodegeneration in cell lines and rodent models. Interestingly, neurodegeneration has not been the only backdrop of chemobrain. There is aberrant neuronal signaling and altered long term potentiation (LTP), suggesting the chemotherapy may not be toxic enough to ensue neurodegeneration, but deleterious enough to impair default neuronal functionality^16^.

The neurological processes of learning and memory storage are reliant on active inter synaptic connectivity strength and subsequently the involvement of larger active synapses will be leading to bigger synaptic efficiency in the form of ‘Long Term Potentiation’ (LTP). Mechanistically and temporally, LTP can be dichotomized into Early LTP (E-LTP), which initiates following inducing stimulus lasting from few minutes to approx. 4 hours, and Late LTP (L-LTP) spanning from few hours to days, dependent on new protein synthesis^17^. The LTP induction and maintenance is stringently managed by ionotropic Glutamate receptors (iGluR)^18^.Aberrant activation of iGluRs may lead to fulminant neuronal death also known as glutamate excitotoxicity^19^. One of the key regulators of LTP are NMDA-R and AMPA-R. These are voltage gated cationic channels with Ca^+2^ and Na^+^ preferences respectively^20^. The case of iGluR being targeted by pharmacologic modulation in order to augment the excitatory neurotransmission, is more than two decades old^21^. The rationale for drug targeting lies in the functional importance of LTP in facilitating learning and memory. The therapeutic benefit can be translated for psychiatric disorders^22^. Therefore, any off target interaction can interfere with NMDA-R transportation and re-sculpting neuronal synapse, thereby rejuvenating synaptic efficiency in general and E-LTP in particular. This resultantly, will affect L-LTP induction which is dependent on E-LTP. The NMDA-R are also known for their critical involvement in neural cell migration and neural tube formation during embryogenesis. So evidently, alteration in their function leads to neural tube defects^23^. Moreover, they have been targeted for therapeutic modulation for CNS disorders like Schizophrenia^24^, Depression^25^, Alzheimer’s disease^26^ and Epilepsy^27^.

The signaling intricacies triggered by LTP induction is not merely linear in its path but rather involve complex interaction and crosstalk of other pathways emaciating into converging or diverging outcome which may end up in positive or negative neuro-signaling feedback loops^17^. Substantial evidence been put forwarded that CICI may be implicated to interfere LTP^15,16^.The investigative ground gets further complicated by considering the cancer patient specific clinico-pathological characteristic and the subsequent combination and dosage of chemotherapy administered^10^. As most of the chemotherapeutic agents have been implicated to affect neurons by more than one mechanism, the resultant outcome of CICI may be attributed to converging and synergistic neurotoxic insults. It is also rather interesting to note that chemotherapy which generally target rapidly dividing cells, can also target very slowly reproducing cells of CNS.

Most of the drugs impart their therapeutic action by stimulating or inhibiting a disease target protein. However, they may be rendered to interact or bind with other proteins i.e. ‘off-target’ due to resemblances in protein binding topological state. These off-target interactions can be a probable eventuality by considering the fact that drugs rarely binds to its only actual target^28^. Such outcome can lead to high drug attrition rate^29^. If an off target is known to mediate a certain side effect, then this information can be potentially utilized to adjust dose, avoid drug side effect and improve management in patients for better clinical outcomes.

The spectrum of neurological deficits by CICI is encompassing almost all classes of chemotherapeutic agents which include alkylating agents, anthracyclines, DNA interfering agents, antitumor antibiotics, mitotic inhibitors, antimetabolites and anti-hormonal agents^30–41^. Paradoxically, the deleterious effects of these agents on brain cortical functions in general and neurodegeneration in particular are well reported^14–16,31,42^, however, the effect of these drugs by virtue of their off target interaction without actually killing neurons but altering their functional dynamics on a particular pathway remains elusive.

The emergence of newer biological, chemical and immunotherapeutic cancer treating agents, with various unconventional drug delivery mechanisms has created scientific plausibility to understand the role of these therapeutic agents and their cumulative outcomes to cell signaling. The advent of kinase inhibitors as chemotherapeutic agents with better blood brain barrier (BBB) permeation^43,44^, intrathecal administration of chemotherapy for managing metastatic disease^45^, and increasing the porosity of BBB by methamphetamine administration^46^, markedly increase the exposure of neuronal microenvironment to chemotherapeutic drugs. It is interesting to note that the employment of *in vivo* experimental tools like yeast two hybrid system and mass spectrometry coupled with tandem affinity purification to experimentally measure protein interactions comes with high false positive rate^47^. Keeping in view of such limitation, the *in silico* prediction tools such as Molecular Docking may provide useful illustration of given 3D drugs structure interactions with large protein datasets. Molecular Docking involves prediction of molecular mechanics among molecules by computation of polyatomic torsional angles, charges and geometry^48^. Docking results in generation of thousands of potential poses of association in which the pose with lowest energy score is predicted to be with best binding mode. The lowest energy scoring reflects binding compactness for a particular ligand conformation bearing a physical or empirical energy function^49^. Although docking is labor intensive, it not simply shows two interactable proteins but also how they interact^50^. Most of the studies on drug target interactions are deploying statistical machine learning algorithms to execute high throughput screening for large drug databases and for genome wide predictions^51–55^. Although such methodology is useful for the intended objective, specific study of actual interactions with the corresponding target protein and the overall implication on the related pathway may be missed which can serve as a useful information for preventing adverse drug reactions. Moreover, the same information can be used for polypharmacology which is already in clinical evaluation with reference to mTOR inhibitors for varying indications such as cancer chemotherapy and Autism spectrum disorders^56–58^.

In spite of off-target interactions by Tamoxifen and its metabolites have been reported^59^, there is a general dearth of information objectively elaborating the underlying biology of side effects causing chemobrain attributed to LTP interference mediated by simultaneous exposure of chemotherapeutic agents. Therefore, this study aimed to understand the drug – protein interaction casted by various chemotherapeutic agents to the major cellular proteins involved in LTP pathway which may impact functionality of these proteins critical for learning and memory processes of brain.

## Experimental Section

**Figure 1:**
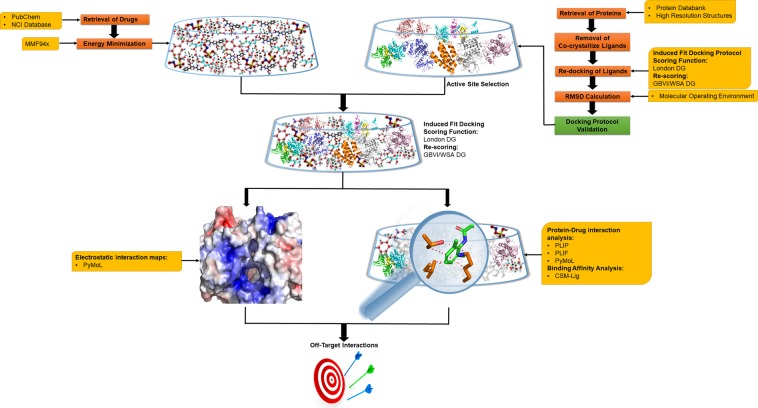
Overall workflow of identification of off-target interactions protocol. Three dimensional structures of NMDA, AMPA, PKA, ERK, CBP and CaMKII were downloaded from protein databank and the structures of chemotherapeutic drugs were obtained from PubChem. These structures were then energy minimized. The validation of docking protocol was done by removal of co-crystallize ligand and re-docking of ligand in molecular operating environment (MOE) followed by RMSD calculation. The docking of drugs with the proteins was then performed using induced fit docking protocol. Top scoring complexes from each protein were then subjected to interaction analysis by protein ligand interaction profiler (PLIP) and PyMOL as well as for electrostatic surface calculations by PyMOL. Binding affinity analysis was done using CSM-Lig server. The ligand interaction fingerprints were calculated using MOE.

A brief workflow used for the identification of off-target interactions between LTP proteins and chemotherapeutic drugs is shown in Fig. 1.

### Selection of drugs

**Figure 2:**
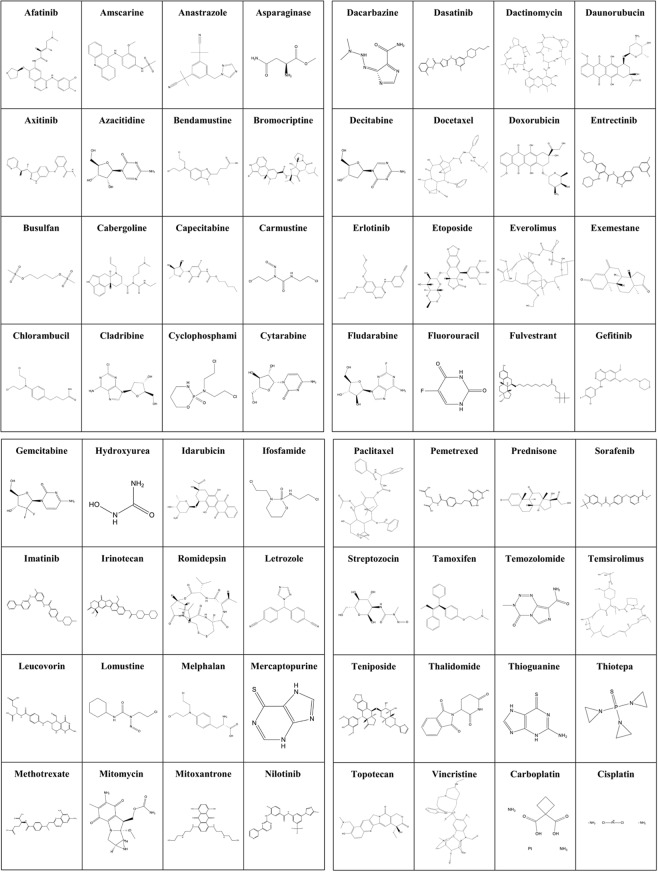
Chemical structure of chemotherapeutic drugs used for identification of off-target interactions.

In order to study the off-target interactions, most commonly used FDA approved chemotherapeutic agents were selected from National Cancer Institute (NCI) directory^60^. Among them are alkylating agents, anti-metabolites, alkaloids, anthracyclines, aromatase inhibitors, nucleoside analogues, anti-hormonal, and antibiotics agents (Fig. 2). The structure of 65 drugs were extracted from PubChem followed by energy minimization using MMF94x force field^61^ implemented in Molecular Operating Environment (MOE) version 2016.08. Database of drugs was then constructed for off-target interaction analysis.

### Selection of proteins to study off-target interactions

**Figure 3:**
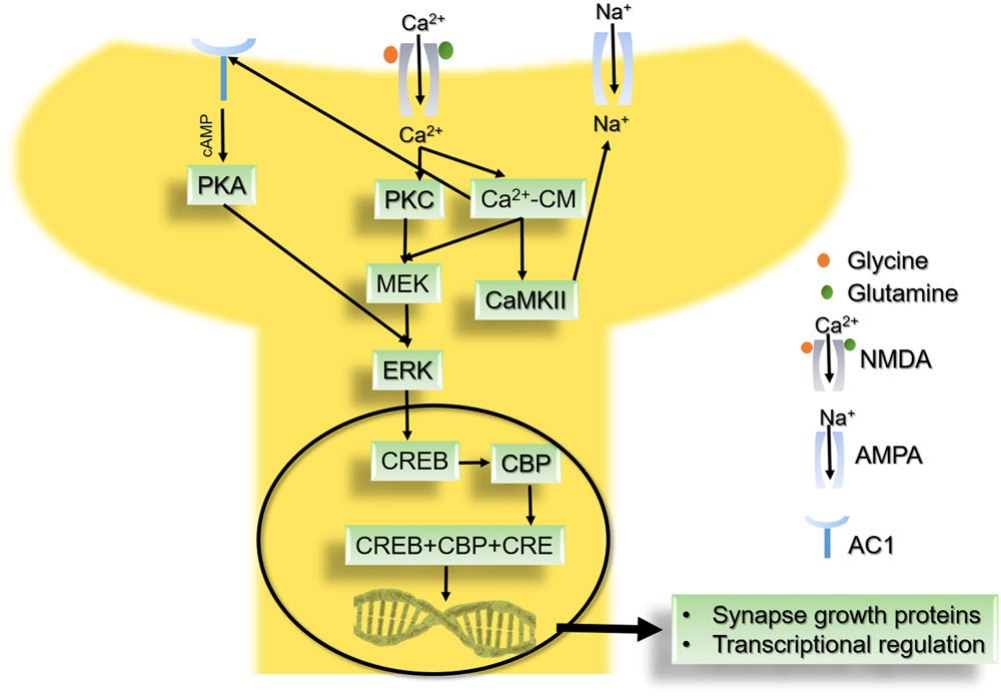
The post synaptic long term potentiation (LTP) pathway^168–170^*. *Adapted from Kegg. (Pathway hsa04270). “Human Long Term Potentiation (LTP)” Retrieved 01-07-2018, from https://www.genome.jp/dbget-bin/www_bget?hsa04720.

In order to study the off-target interactions of chemotherapeutic drugs on the cognition, long term potentiation pathway was selected (Fig. 3). The major regulators of LTP pathway are *N*-methyl-D-aspartate receptor (NMDA), α-amino-3-hydroxy-5-methyl-4-isoxazolepropionic acid receptor (AMPA), Ca^2+^/calmodulin-dependent protein kinase II (CaMKII), protein kinase A (PKA), CREB-binding protein, and extracellular signal–regulated kinase (ERK).

Structurally, NMDARs are comprised of dimers of N subunits (N 1 & 2), which are linked together to form tetramers in a homo/hetero dimer fashion^62,63^. Each N subunit is harboring an amino-terminal domain (ATD), followed by a Ligand Binding Domain (LBD), Transmembrane Domain and finally the C-Terminal Domain (CTD) oriented intracellularly^64^. The ligand binding domain of NMDA composed of two subunits NR1 and either NR2A/B/C or D. Our study involved NR2A which is further composed of S1 (462–502 a.a) and S2 (664–720 a.a) similarly NR1 is contributing to LBD involved residues of S1(480–526 a.a) and S2 (682–740 a.a) subunits.

In case of AMPA, the ligand binding domain falls into S1 (393–506 a.a) and S2 (632–733 a.a) domain. In case of AMPA the ZK1 antagonist bound to the ligand binding domain.

CaMKII composed of kinase domain (1–273 a.a), the regulatory segment (274–314 a.a), and hub domain (315–475 a.a). Regulatory segment further composed of R1 (274–291 a.a), R2 (292–297 a.a) and R3 (298–314 a.a) subunits. The CaM binding residues span residues 290 to 314 within this segment. Any ligand/ inhibitor bound to the regulatory subunit alter the function of CaMKII (A mechanism of tunable autoinhibition).

PKA functions with the help of 2 regulatory and 2 catalytic subunits, assembling into a tetrameric holoenzyme. PKA consists of CaMKII binding domain-A and B (CBD-A and CBD-B). the R1α comprises both CBDs with sequence from 91–379 a.a. Within this region there are several sub domains with major allosteric or binding hot spots include, the N3A motif (residues 119–150 a.a), the β2–3 loop (residues 163–171 a.a), the base binding region (BBR) (residues 180–193 a.a), the phosphate binding cassette (PBC) (residues 199–211 a.a), and the hinge (residues 226–251 a.a). Another important domain in the PKA is the glycine rich loop (50–55 a.a) and the ribose binding pocket^65,66^.

Human ERK2 is spanning on 360 amino acids. Structurally they are similar to other kinases which constitutes protein kinase domain (25–313 aa)^67^. This domain harbors α and β helices which further host glycine rich loop (32–37 a.a), hinge region (106–109) and ATP phosphate binding loop^68^.

Computational studies on human CREB are scarcely available probably because of its unstructured and intrinsically disordered behavior in solution^69,70^. This can render interaction prediction against CREB with computational inaccuracies. Therefore, we restricted our docking analysis with CBP which itself is harboring intrinsically disordered regions.

Human CREB binding protein (CBP) comprises of 2442 amino acids, almost 50% of which is reported to be intrinsically disordered. CBP constitutes a histone acetyl transferase (HAT) domain, bromo domain (BRD), KID binding domain (KIX), plant homeodomain (PHD) and transcriptional activator zinc finger (TAZ) domain^71–73^.

### Protein structure preparation

The atomic coordinates of proteins, NMDA (pdb ID = 5KDT), AMPA (pdb ID = 5KBV), CaMKII (pdb ID = 3SOA), PKA (pdb ID = 4UJA), CBP (pdb ID = 4NR5), and ERK (pdb ID = 2OJJ), were retrieved from Protein Data Bank. The details of all the structures are shown in Table 1. Proteins retrieved from the Pdb contained water molecules and also the original ligand/substrate/inhibitor. Thus, for the preparation of the proteins structures for ligand docking, co-crystallized ligand and any water molecules present were removed followed by protonation and energy minimization using AMBER 99 force field in MOE.

**Table 1:** List of proteins used in the study for docking analysis.

Protein	PDB ID	Resolution (Å)	Structure Title	Ligand	Specie	Ref
NMDA	5KDT	2.44	Structure of the human GluN1/GluN2A LBD in complex with GNE0723	(1~{R},2~{R})-2-[7-[[5-chloranyl-3-(trifluoromethyl)pyrazol-1-yl]methyl]-5-oxidanylidene-2-(trifluoromethyl)-[1,3]thiazolo[3,2-a]pyrimidin-3-yl]cyclopropane-1-carbonitrile	Homo sapiens	^171^
AMPA	5KBV	6.8	Cryo-EM structure of GluA2 bound to antagonist ZK200775 at 6.8 Angstrom resolution	{[7-morpholin-4-yl-2,3-dioxo-6-(trifluoromethyl)-3,4-dihydroquinoxalin-1(2H)-yl]methyl}phosphonic acid	Rattus norvegicus	^75^
ERK	2OJJ	2.4	Crystal structure of ERK2 in complex with (S)-N-(1-(3-chloro-4-fluorophenyl)-2-hydroxyethyl)-4-(4-(3-chlorophenyl)-1H-pyrazol-3-yl)-1H-pyrrole-2-carboxamide	(s)-n-(1-(3-chloro-4-fluorophenyl)-2-hydroxyethyl)-4-(4-(3-chlorophenyl)-1h-pyrazol-3-yl)-1h-pyrrole-2-carboxamide	Homo sapiens	^78^
PKA	4UJA	1.93	Protein Kinase A in complex with an Inhibitor	7-{(3S,4R)-4-[(5-bromothiophen-2-yl)carbonyl]pyrrolidin-3-yl}quinazolin-4(3 H)-one	Homo sapiens	^77^
CBP	4NR5	1.66	Crystal structure of the bromodomain of human CREBBP in complex with an isoxazolyl-benzimidazole ligand	5-(3,5-dimethyl-1,2-oxazol-4-yl)-1-[2-(morpholin-4-yl)ethyl]-2-(2-phenylethyl)-1H-benzimidazole	Homo sapiens	To be published
CaMKII	3SOA	3.5	Full-length human CaMKII	4-[(2,4-dichloro-5-methoxyphenyl)amino]-6-methoxy-7-[3-(4-methylpiperazin-1-yl)propoxy]quinoline-3-carbonitrile	Homo sapiens	^76^

### Docking protocol

The docking studies were performed using induced fit docking (IFD) protocol implemented in MOE version 2016.08^74^. For each docking run, the active site was identified on the basis of interaction of co-crystallized ligand with each protein. The co-crystallize ligand 6RV had been bound to the ligand binding domain of NMDA. For docking the 5 Å area around the interacting residues of NMDA (S1 and S2 domain) with 6RV was selected as active site (Volgraf *et al*. 2016). In AMPA, the co-crystallize ligand had been ZK1 bound at the ligand binding domain. A 5 Å area around the important residues that involved in interaction with ZK1 was selected as active site for docking of library of compounds^75^. In case of CaMKII, the bound inhibitor had been Bosutinib, targeting its regulatory domain, hence the 5 Å area around the Bosutinib binding pocket was selected as active site for further docking studies^76^. In case of PKA, the inhibitor 4L7 had been bound to glycine rich loop and β2–3 loop (ribose pocket). The 5 Å area around the residues involved in interactions with 4L7 was used as active site for docking studies^77^. In ERK2, the co-crystallize ligand had been 82 A that bound to protein kinase and glycine rich loop. A 5 Å area around the protein kinase and glycine rich loop was selected as active site for docking of compound’s library^78^. In case of CBP, the bound ligand had been 2LL attached to its bromo domain. The 5 Å area around the important residues that involved in interactions was selected for docking studies. The docking calculation were performed using triangle match as placement method with London DG as scoring function and re-scoring was performed with GBVI/WSA dG^79,80^. This was followed by ranking of the lowest energy protein-ligand interaction poses. The complexes with most negative IFD scores were considered carrying favorable binding. The validation step of docking protocol was performed through re-docking of the same default ligand with RMSD calculations. After re-docking, the RMSD value of co-crystallized and re-docked ligand was calculated. After the validation of docking protocol through re-docking, the library of chemotherapeutic compounds were docked into the binding domains of NMDA, AMPA, PKA, CBP, CaMKII, and ERK using the same protocol. The protocol generated 30 conformational poses for each drug with all selected proteins. The poses were re-scored by using GBVI/WSA Dg scoring function.

### Binding affinity analysis

The scoring analysis of each protein with the studied drugs was performed using box-plot function in R-3.3.3 package. On the basis of docking scores, top five complexes for each protein with studied drugs were selected for interaction analysis. The interaction analysis was performed using protein ligand interaction profiler (PLIP) server^81^ and PyMOL (PyMOL, Molecular Graphics System, Version 2.0 Schrodinger, LLC). To further verify the interactions between docked complexes, protein ligand interaction fingerprints (PLIF) were calculated using PLIF algorithm implemented in MOE^79,80^. PLIF summarizes the interactions like H-bonds, ionic and surface contacts on the basis of fingerprint scheme that is representative of ligand-protein complex^79,80^. In order to further probe off-target interactions by top scoring chemotherapeutic drugs, the binding affinity of the top scoring docked complexes were calculated using CSM-Lig^82^. CSM-Lig predict the binding affinity of a protein-small molecule complex based on structural signatures and machine learning algorithm^82^.

### Physicochemical attributes of binding region

The three dimensional illustration of charge distribution among molecules is depicted by Electrostatic potential maps, also known as electrostatic potential energy maps. These maps aid in determination of variably charged regions of a molecule which can help in identifying intermolecular interactions and molecular properties of small molecules^83^. To understand the binding surfaces of NMDA, AMPA, PKA, CBP, CaMKII, and ERK, electrostatic charge distribution were studied using APBS plugins in PyMol.

## Results

### Interaction analysis with LTP proteins

The off-targets of drugs were identified on the basis of docking scores (lower the scores, strong is the interactions).

**Figure 4:**
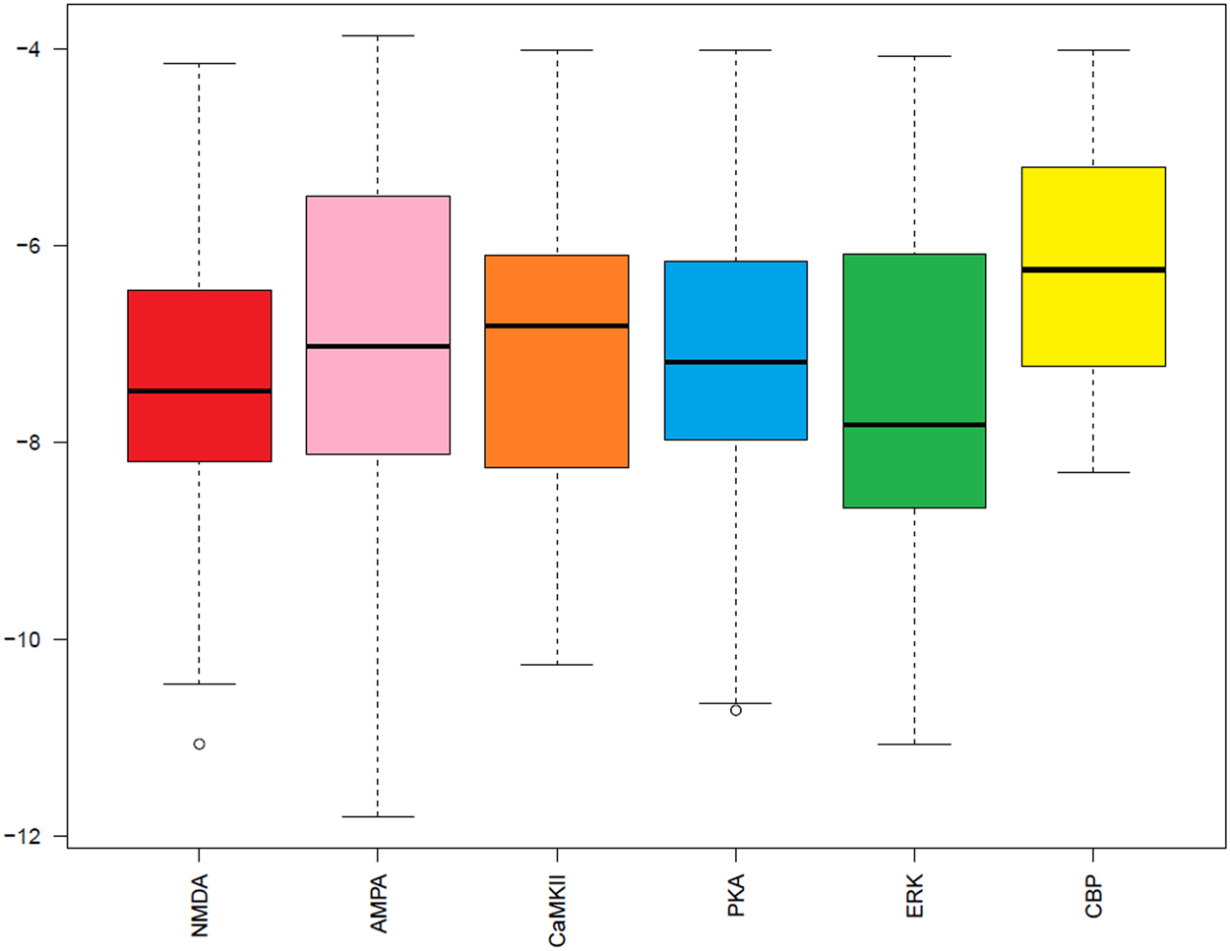
Box plot of docking scores generated by MOE. Y-axis represent the scores while X-axis represent the name of proteins. NMDA *(N*-methyl-D-aspartate receptor), AMPA (α-amino-3-hydroxy-5-methyl-4-isoxazolepropionic acid receptor), CaMKII (Ca^2+^/calmodulin-dependent protein kinase II), PKA (protein kinase A), ERK (extracellular signal–regulated kinase), and CBP (CREB-binding protein).

The docking scores of all the studied protein are presented in the form of box plot (Fig. 4). According to box plot, the ERK protein (a protein kinase) is having the median score of −7.8 with 75% of data in the upper quartile and 25% of data in the lower quartile. The median score of NMDA (a receptor protein) is −7.4 with 75% of data in upper quartile and 25% of data in lower quartile. PKA is a protein kinase with median score of −7.1 with 70% and 30% of data in upper and lower quartile, respectively. Another receptor protein of LTP pathway is AMPA having the median score of −7.0. In AMPA, 70% of data is present in upper quartile and 30% of data is in lower quartile. CaMKII, a kinase protein is having different distribution of data with 25% of data is in upper quartile and 75% of data is in lower quartile with median score of −6.8. CBP is a nuclear protein with the highest median value of −6.2 and with equal distribution of data in both quartiles.

**Figure 5:**
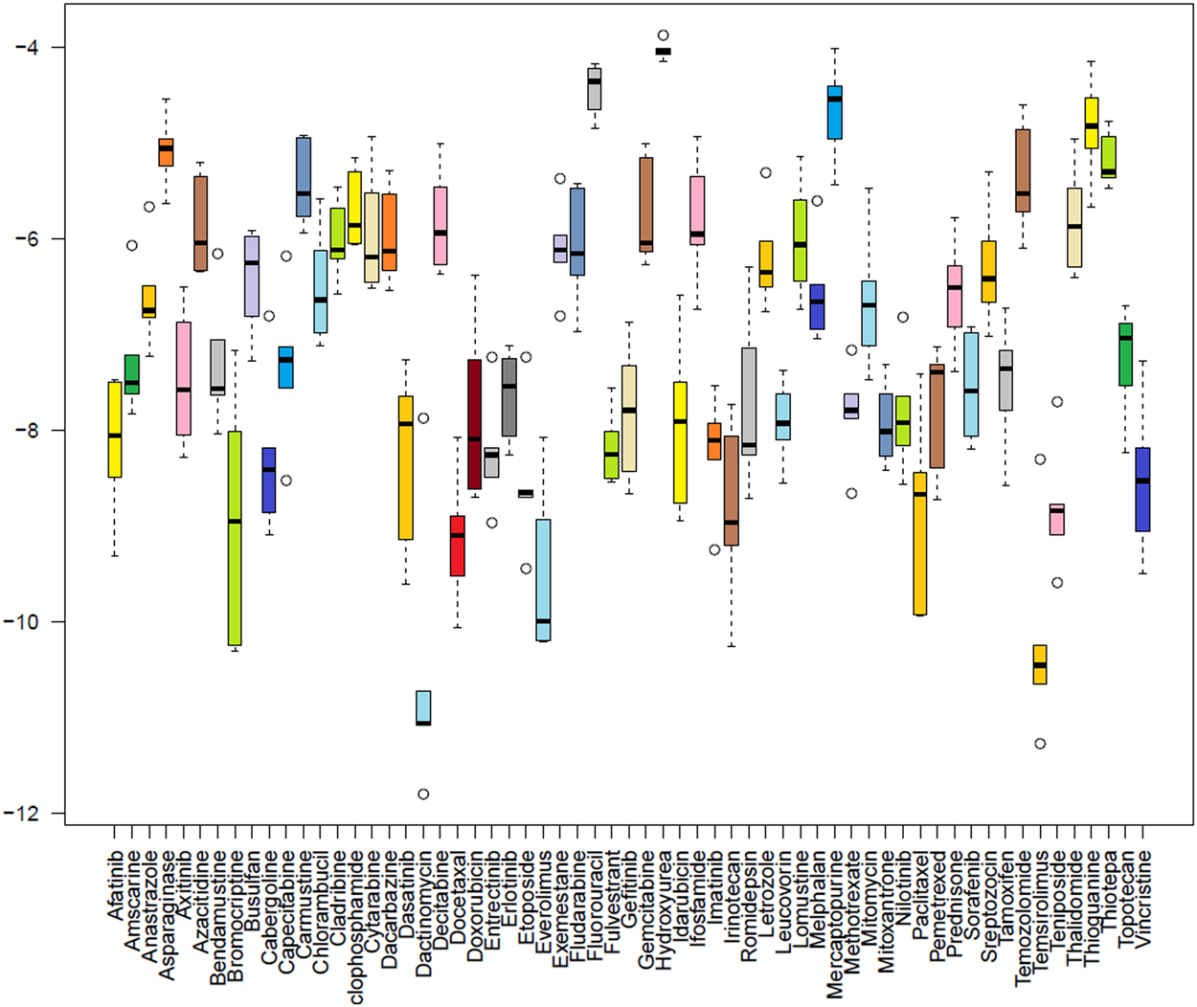
Box plot of docking scores generated by MOE. Y-axis represent the scores while X-axis represent chemotherapeutic drugs. Dactinomycin, temsirolimus and Everolimus are the drugs that are having minimum docking scores.

The scores of all the chemotherapeutic drugs on the basis of their interactions with all the studied proteins is shown in Fig. 5. According to median values, Dactinomycin is having the lowest median scores of −10.8 with 100% of data present in upper quartile. Temsirolimus is having a median score of −10.3 with almost equal data distribution in both the quartile. Everolimus is having the median score of −9.7 with 15% of data in lower quartile and 85% of data in upper quartile. Bromocriptine and Docetaxel are having the same median score of −9.0 but with different distribution of data. In Bromocriptine, 60% of data in the lower quartile and 40% of data in upper quartile while in Docetaxel 75% of data in lower quartile and 25% of data in the upper quartile. Teniposide and Irinotecan having the median scores of −8.9 and −8.8, respectively. In, Teniposide 5% of data in upper quartile and 95% of data in lower quartile while in Irinotecan 85% of data in lower quartile and 15% of data in upper quartile. Paclitaxel, and Etoposide are having the median score of −8.6 but with different distribution of data. In paclitaxel 10% of data is in upper quartile and 90% of data is in lower quartile, while etoposide 100% of data present at the median. The median score of Afatinib is −8.4 with equal distribution of data in both quartiles. The drugs with the median scores in the range of −5 to −6 are Asparaginase, Busulfan, Carmustine, Cladribine, Cyclophosphamide, Cytarabine, Dacarbazine, Decitabine, Exemestane, Fludarabine, Gemcitabine, Ifosfamide, Letrozole, Lomustine, Melphalan, Mitomycin, Prednisone, Streptozocin, Temzolomide, Thalidomide, Thioguanine, and Thiotepa. The drugs with median scores above −4.5 are Fluorouracil, Hydroxyurea, Mercaptopurine.

#### Interaction of Drugs with NMDA

**Figure 6:**
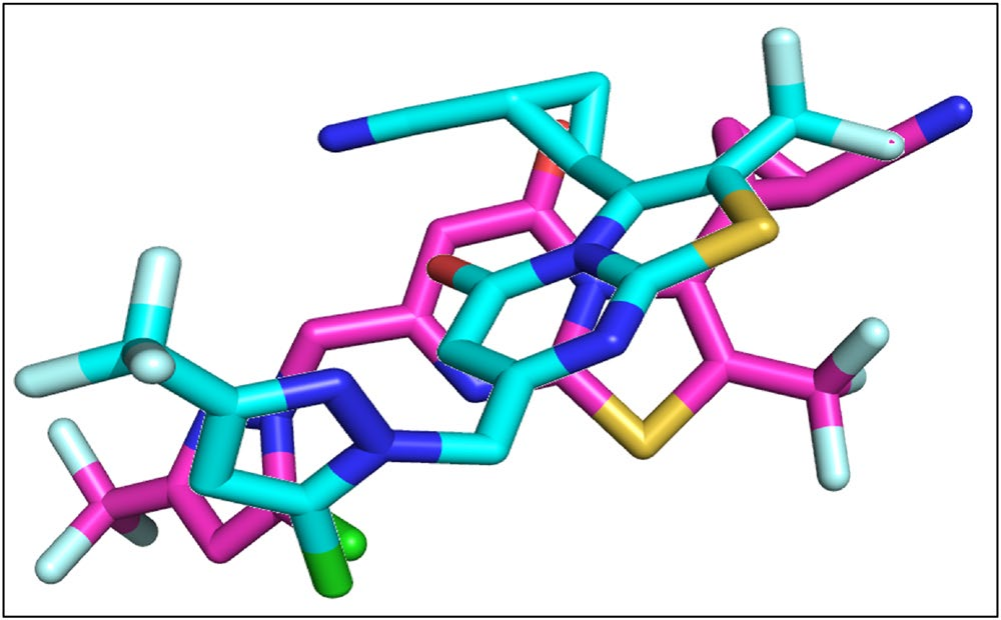
The re-docked pose of 6RV. The co-crystallized ligand is shown in cyan while the re-docked ligand is shown in purple.

**Figure 7:**
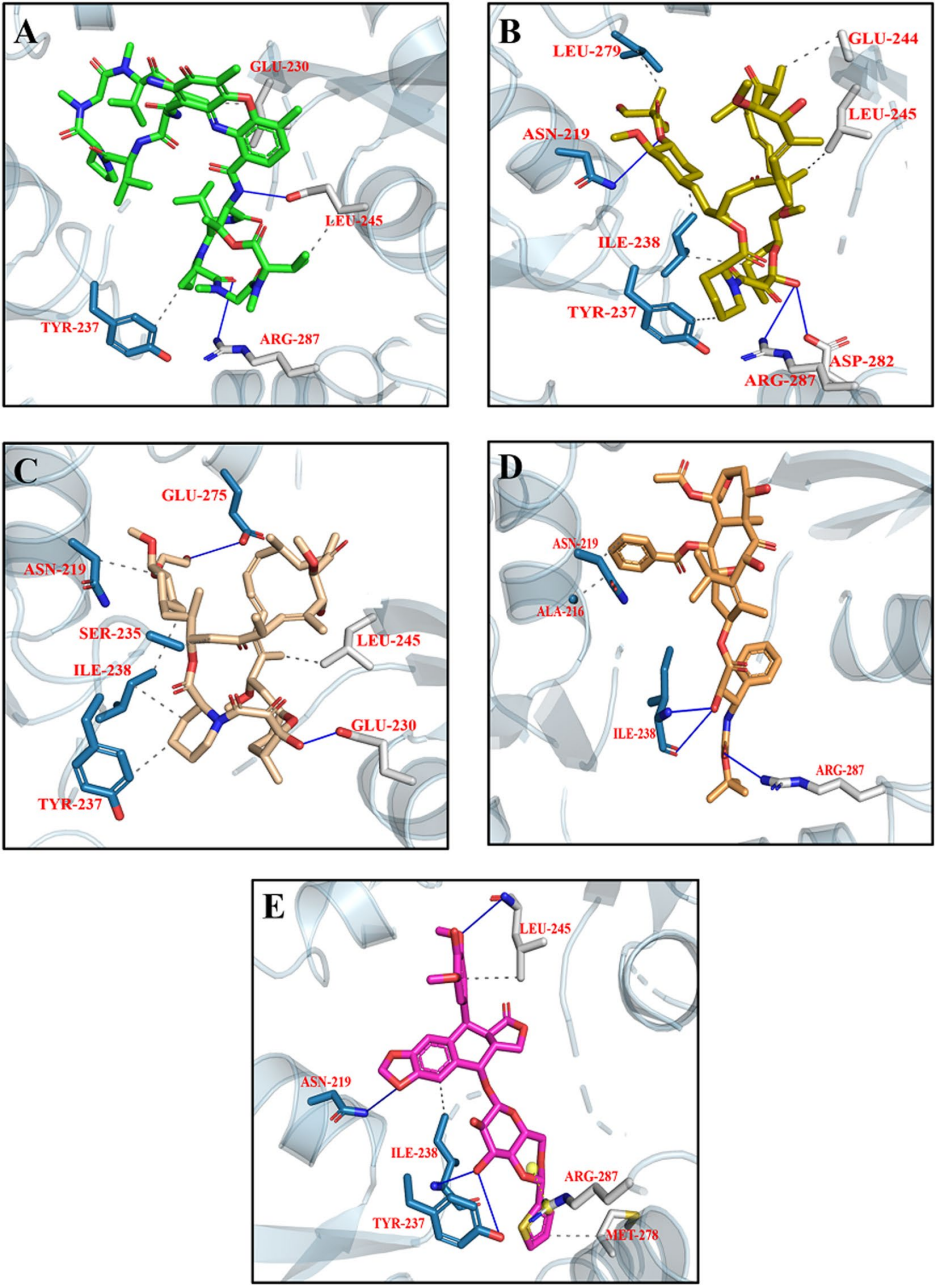
Top scoring docking conformation of NMDA with (**A**) Dactinomycin (green); (**B**) Temsirolimus (yellow); (**C**) Everolimus (beige); (**D**) Docetaxel (golden); and (**E**) Teniposide (purple). Glu N1 residues shown in white while Glu N2A residues shown in blue.

The re-docking of co-crystallize ligand was performed into the binding pocket of NMDA with overall score of −7.3 Kcal/mol and RMSD value 3.8 Å. Figure 6 is showing the original and re-docked conformation of 6RV into the binding pocket of NMDA. After the successful re-docking, the docking of library of compounds were performed using the same protocol. For each compound, 30 conformations were explored and the top scoring docking poses of each compound were further used for studies. The details of docking scores of each compound is shown in Supplementary Fig. S1. Top five docking complexes were further evaluated for ligand protein interactions (Fig. 7). The docking scores of Dactinomycin, Temsirolimus, Everolimus, Docetaxel, and Teniposide are considerably lower in comparison to that of the bound inhibitor (6RV), thus displaying the superior binding affinity of chemotherapeutic drugs to NMDA. Dactinomycin binds with a score of −11, Temsirolimus with −10.4, Everolimus with −10.2, docetaxel with −8.9, and Teniposide with −8.8. The docking scores were further validated by calculating ligand binding affinities of top five complexes. Ligand binding affinities are corresponding to the docking scores. The drug with the highest docking score is predicted to be with greater affinity for NMDA. Dactinomycin is having the highest binding affinity for NMDA with energy value of 37.0. Temsirolimus and Everolimus are having the binding affinity values of 31.3 and 31.1, respectively. All five drugs have shown interactions with Tyr 237 A, Leu 245B, and ILE 238 A, involved in hydrophobic interactions while Arg 287B involved in H-bonding (Table 2).

**Table 2:** Interacting residues of NMDA with Dactinomycin, Temsirolimus, Everolimus, Docetaxel, and Teniposide.

Drugs	Binding Affinity (−log_10_(K_D_|K_i_))	Protein Residue	Distance (Å)	Type of Interactions
Dactinomycin	37.0	GLU 230B	3.61	Hydrophobic
		TYR 237A	3.74	Hydrophobic
		LEU 245B	3.61	Hydrophobic
		GLU 230B	2.20	H-bond
		ARG 287B	3.60	H-bond
Temsirolimus	31.1	TYR 237A	3.94	Hydrophobic
		ILE 238A	3.80	Hydrophobic
		GLU 244B	3.73	Hydrophobic
		LEU 245B	3.50	Hydrophobic
		LEU 279A	3.82	Hydrophobic
		ASN 219A	3.44	H-bond
		ASP 282B	2.53	H-bond
		ARG 287B	3.50	H-bond
Everolimus	31.3	ASN 219A	3.79	Hydrophobic
		SER 235A	3.66	Hydrophobic
		TYR 237A	3.32	Hydrophobic
		ILE 238A	3.94	Hydrophobic
		LEU 245B	3.09	Hydrophobic
		GLU 230B	1.63	H-bond
		LEU 245B	3.27	H-bond
		GLU 275A	3.11	H-bond
Docetaxel	29.4	ALA 216A	3.59	Hydrophobic
		ASN 219A	3.86	Hydrophobic
		ILE 238A	2.65	H-bond
		ARG 287B	2.34	H-bond
Teniposide	−45.2	ILE 238A	3.97	Hydrophobic
		LEU 245B	3.76	Hydrophobic
		MET 278B	3.70	Hydrophobic
		ASN 219A	3.25	H-bond
		TYR 237A	3.25	H-bond
		ILE 238A	2.30	H-bond
		LEU 245B	2.89	H-bond

#### Interaction of Drugs with AMPA

**Figure 8:**
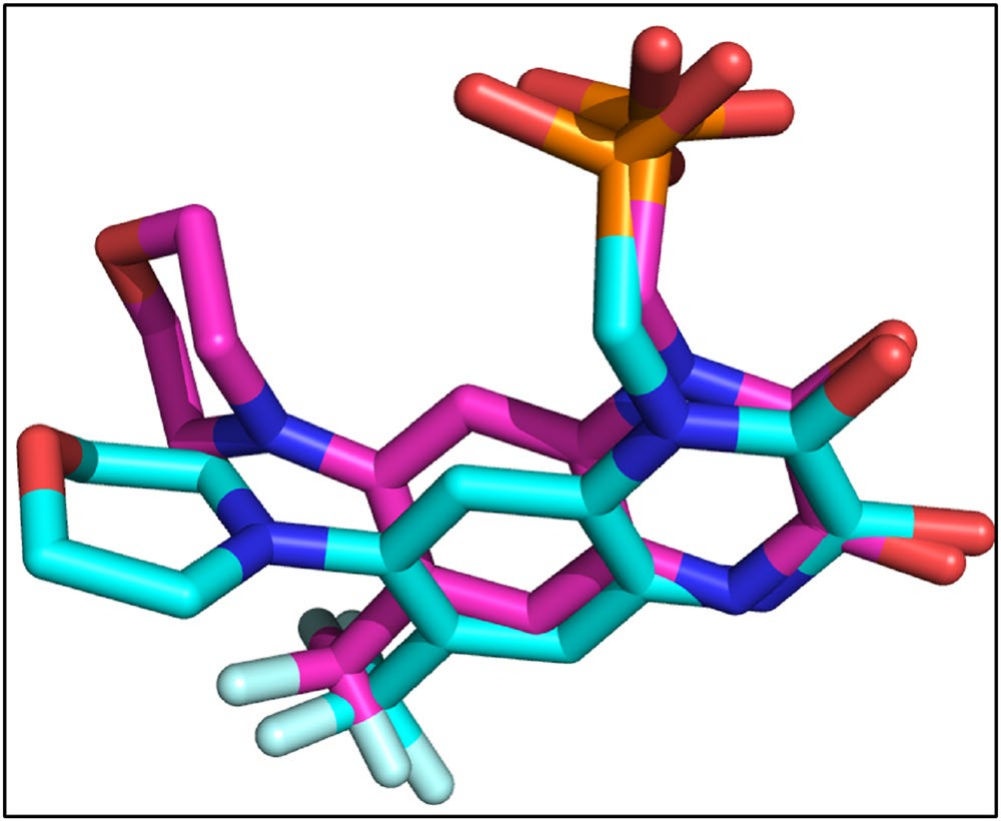
The re-docked conformation of ZK1. The co-crystallized ligand is shown in cyan while the re-docked ligand is shown in purple.

**Figure 9:**
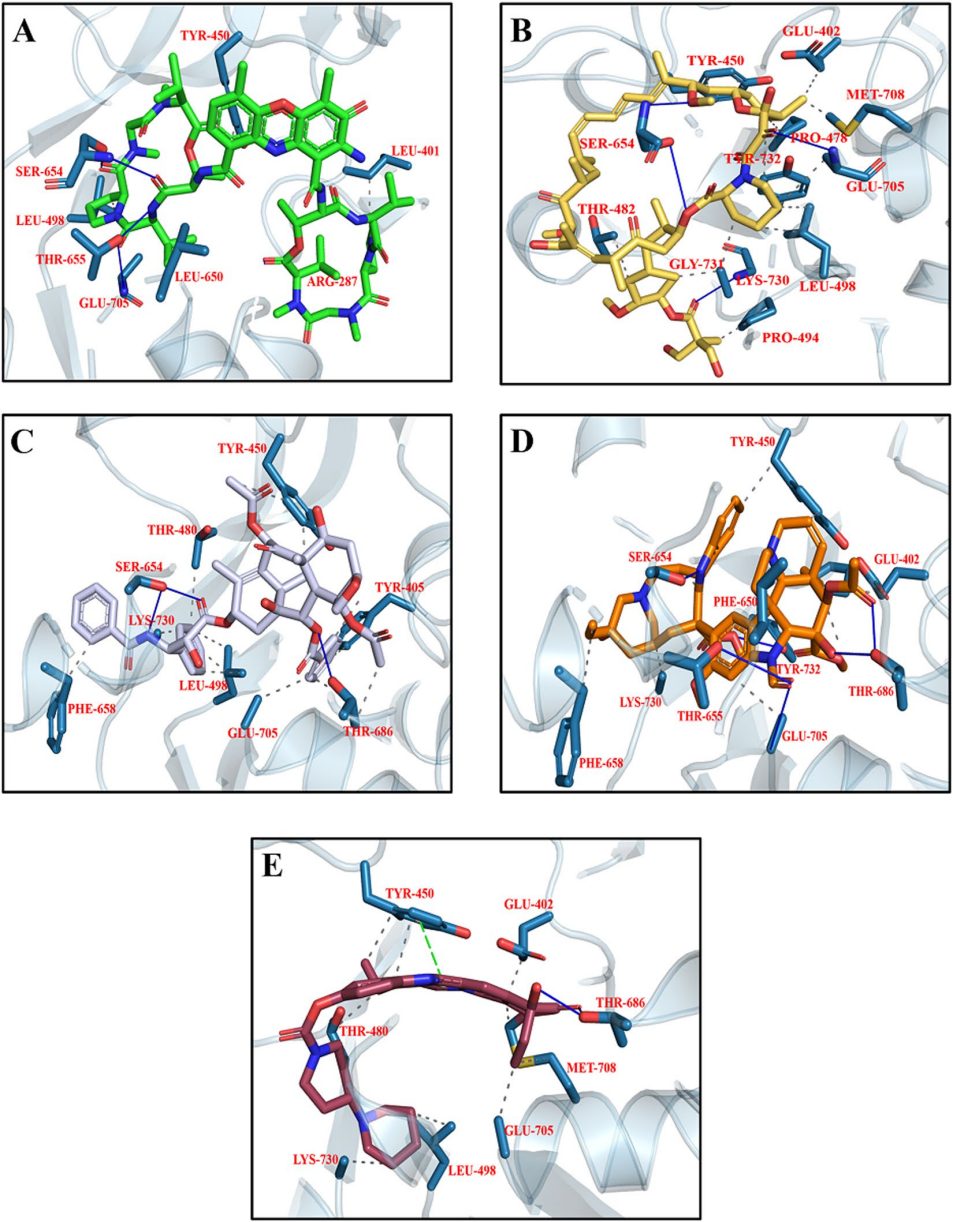
Top ranking docking poses of AMPA with (**A**) Dactinomycin (green); (**B**) Temsirolimus (yellow); (**C**) Paclitaxel (silver); (**D**) Vincristine (golden); and (**E**) Irinotecan (maroon).

The validity of docking protocol was done through re-docking of ZK1 (co-crystallize ligand) into the active site of AMPA. ZK1 was re-docked with energy value of −6.99 Kcal/mol and RMSD of 1.5 Å (Fig. 8). Using the same docking protocol, the docking of our library of compounds into the ligand binding domain (S1 (393–503 a.a) and S2 (632–773 a.a)) of AMPA, were performed. For each compound 30 different docking conformations were generated (Fig. S2) and the top scoring docking conformations were further explored for ligand binding interactions. The ligands bind to the ligand binding domain with a much higher affinity compared to that of the allosteric site. This is shown by the differences in docking scores. In the ligand binding domain, the co-crystallize inhibitor binds with a binding strength of −6.99 Kcal/mol. While Dactinomycin, Temsirolimus, Paclitaxel, Vincristine, and Irinotecan binds with scores of −11.8, −11.2, −9.9, −9.5, and −9.1 Kcal/mol, respectively. The ligand binding affinities are comparable to the docking scores with Temsirolimus is having highest affinity for AMPA and Irinotecan is the least. Individual ligand binding interactions are shown in Fig. 9 and Table 3. All five drugs showing the hydrophobic interactions with Tyr450 and Leu 498 while H-bonding with Ser 654 and Glu 705.

**Table 3:** Interacting residues of AMPA with Dactinomycin, Temsirolimus, Paclitaxel, Vincristine, and Irinotecan.

Drugs	Binding Affinity (−log_10_(K_D_|K_i_))	Protein Residue	Distance (Å)	Type of Interactions
Dactinomycin	37.1	LEU 410	3.91	Hydrophobic
		TYR 450	3.29	Hydrophobic
		LEU 498	2.54	Hydrophobic
		LEU 650	3.87	Hydrophobic
		SER 654	2.75	H-bond
		THR 655	2.13	H-bond
		GLU 705	2.80	H-bond
Temsirolimus	38.2	GLU 402	3.90	Hydrophobic
		TYR 450	3.65	Hydrophobic
		PRO 478	478	Hydrophobic
		THR 482	3.93	Hydrophobic
		PRO 494	2.99	Hydrophobic
		LEU 498	2.78	Hydrophobic
		GLU 705	3.77	Hydrophobic
		MET 708	2.32	Hydrophobic
		LYS 730	3.30	Hydrophobic
		TYR 732	3.85	Hydrophobic
		SER 654	2.82	H-bond
		GLU 705	2.98	H-bond
		GLY 731	2.89	H-bond
Paclitaxel	36.1	TYR 405	3.03	Hydrophobic
		TYR 450	3.80	Hydrophobic
		THR 480	3.56	Hydrophobic
		LEU 498	3.66	Hydrophobic
		PHE 658	3.71	Hydrophobic
		GLU 705	3.29	Hydrophobic
		LYS 730	3.00	Hydrophobic
		SER 654	2.42	H-bond
		THR 686	2.77	H-bond
Vincristine	34.4	GLU 402	3.56	Hydrophobic
		TYR 450	3.11	Hydrophobic
		LEU 650	3.38	Hydrophobic
		PHE 658	3.99	Hydrophobic
		LYS 730	3.01	Hydrophobic
		SER 654	1.74	H-bond
		THR 655	3.15	H-bond
		THR 686	1.87	H-bond
		GLU 705	2.66	H-bond
		TYR 732	2.95	H-bond
Irinotecan	35.1	GLU 402	2.71	Hydrophobic
		TYR 450	3.03	Hydrophobic
		THR 480	3.47	Hydrophobic
		LEU 498	3.35	Hydrophobic
		GLU 705	3.02	Hydrophobic
		MET 708	2.22	Hydrophobic
		LYS 730	3.55	Hydrophobic
		THR 686	1.80	H-bond
		TYR 450	3.52	Pi-stacking

#### Interaction of Drugs with PKA

**Figure 10:**
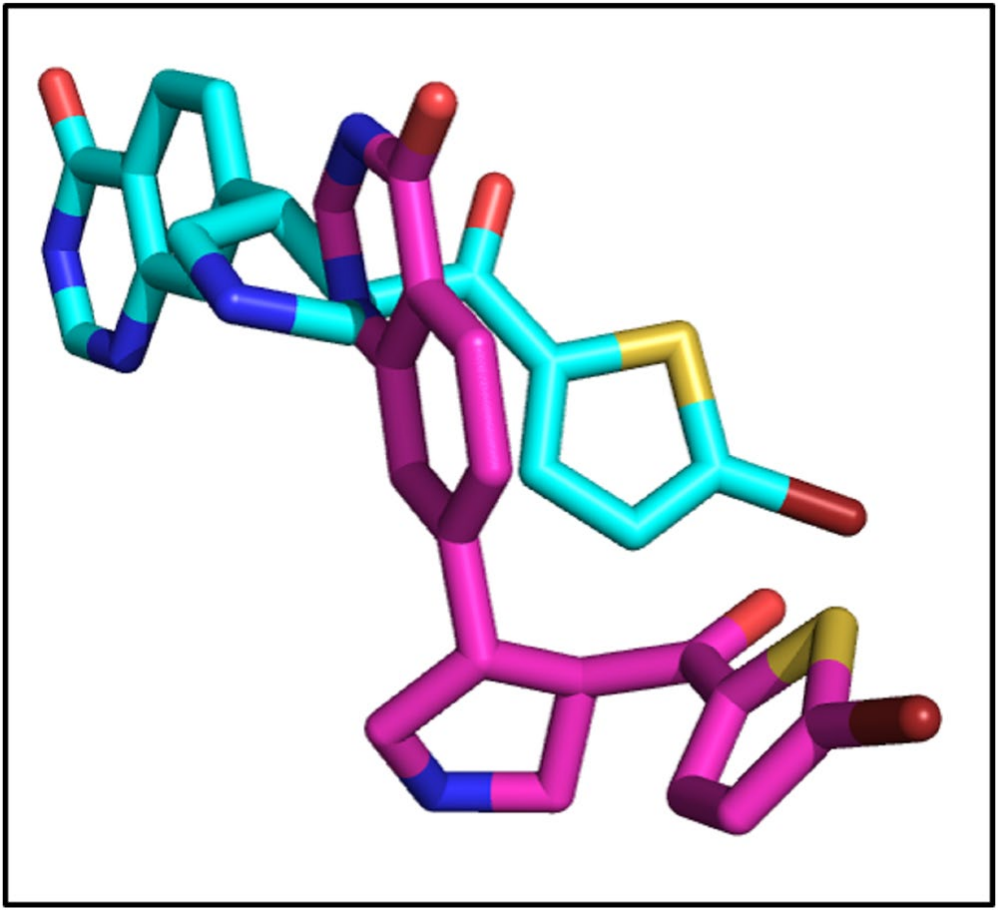
The original and re-docked conformation of 4L7. The co-crystallized ligand is shown in cyan while the re-docked ligand is shown in purple.

The crystal structure of PKA was retrieved with 4L7 as co-crystallized ligand. 4L7 was re-docked into the binding pocket of PKA with binding affinity of −6.1 Kcal/mol (Fig. 10).

**Figure 11:**
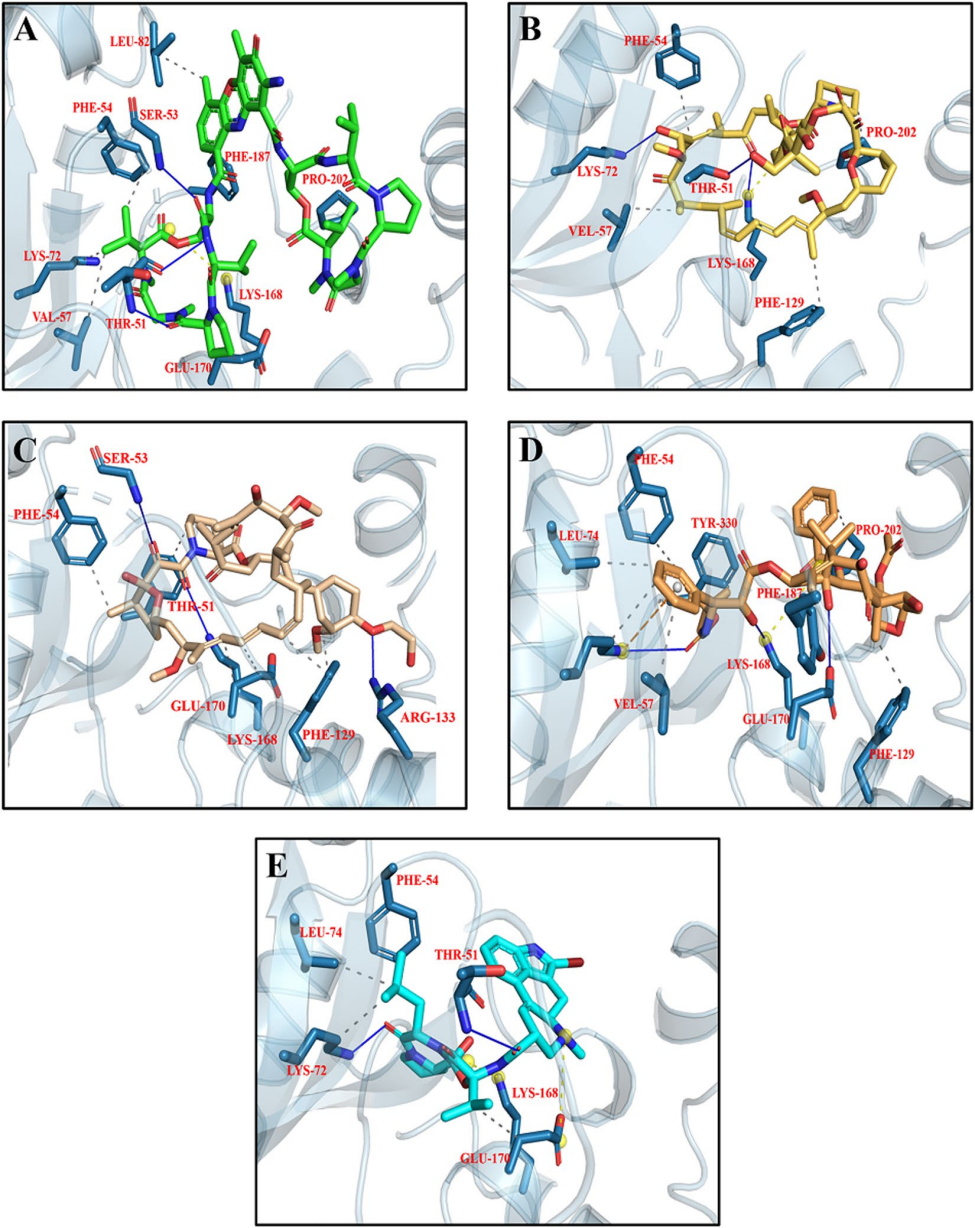
Top five docking conformations of PKA with (**A**) Dactinomycin (green); (**B**) Temsirolimus (yellow); (**C**) Everolimus (beige); (**D**) Docetaxel (golden); and (**E**) Bromocriptine (cyan).

The library of chemotherapeutic drugs were docked into the binding pocket of PKA and 30 conformations per compound were generated. The detail of docking scores of all the compounds is shown in Fig. S3. Among all the docked conformations, top five docking complexes were further studied for ligand binding interactions (Fig. 11; Table 4). On the basis of docking scores, it has been observed that the studied drugs are having better affinity for PKA compared to co-crystallized ligand. Dactinomycin, Temsirolimus, Everolimus, Docetaxel and Bromocriptine bind with the PKA with scores of −10.7, −10.6, −9.7, −9.5, and −9.3 Kcal/mol, respectively. Ligand binding affinities of top five complexes are shown in Table 4. Dactinomycin is having the highest binding affinity for PKA with score of 39.1 while bromocriptine is having the least binding affinity for PKA. All the five drugs having hydrophobic interactions with Phe 54, Val 57, and H-bonding with Thr 51 in the glycine rich loop of PKA. In β2–3 loop, Lys 168 involved in either H-bonding or formed salt bridge with ligand atoms. In phosphate binding cassette, Pro 202 also involved in hydrophobic interactions.

**Table 4:** Interacting residues of PKA with Dactinomycin, Temsirolimus, Everolimus, Docetaxel, and Bromocriptine.

Drugs	Binding Affinity (−log_10_(K_D_|K_i_))	Protein Residue	Distance (Å)	Type of Interactions
Dactinomycin	39.1	PHE 54	3.03	Hydrophobic
		VAL 57	3.92	Hydrophobic
		LYS 72	3.55	Hydrophobic
		LEU 82	3.95	Hydrophobic
		GLU 170	3.57	Hydrophobic
		PHE 187	3.74	Hydrophobic
		PRO 202	3.93	Hydrophobic
		THR 51	2.12	H-bond
		SER 53	2.34	H-bond
		LYS 168	5.13	Salt bridge
Temsirolimus	35.5	PHE 54	3.35	Hydrophobic
		VAL 57	3.32	Hydrophobic
		PHE 129	3.26	Hydrophobic
		PRO 202	3.72	Hydrophobic
		THR 51	2.47	H-bond
		LYS 72	2.33	H-bond
		LYS 168	2.56	H-bond
		LYS 168	5.48	Salt bridge
Everolimus	37.3	PHE 54	3.21	Hydrophobic
		PHE 129	3.43	Hydrophobic
		GLU 170	3.72	Hydrophobic
		THR 51	3.96	Hydrophobic
		SER 53	3.28	H-bond
		ARG 133	1.94	H-bond
		LYS 168	3.18	H-bond
Docetaxel	38.2	PHE 54	3.25	Hydrophobic
		VAL 57	3.52	Hydrophobic
		LEU 74	3.52	Hydrophobic
		PHE 129	3.21	Hydrophobic
		PHE 187	3.34	Hydrophobic
		PRO 202	3.73	Hydrophobic
		TYR 330	3.84	Hydrophobic
		LYS 168	2.39	H-bond
		GLU 170	3.17	H-bond
		LYS 72	4.87	Pi-stacking
		LYS 168	4.86	Salt bridge
Bromocriptine	33.8	PHE 54	3.78	Hydrophobic
		LYS 72	3.45	Hydrophobic
		LEU 74	3.52	Hydrophobic
		GLU 170	3.84	Hydrophobic
		THR 51	2.66	H-bond
		LYS 72	1.59	H-bond
		LYS 168	3.70	Salt bridge
		GLU 170	5.34	Salt bridge

#### Interaction of Drugs with CaMKII

**Figure 12:**
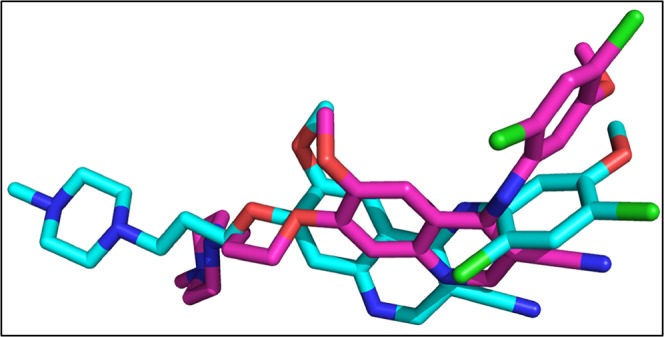
The co-crystallize and re-docked conformation of Bosutinib. The co-crystallized ligand is shown in cyan while the re-docked ligand is shown in purple.

The co-crystallize ligand into the binding pocket of CaMKII is Bosutinib present in the regulatory domain of CaMKII. The Bosutinib was re-docked into the binding domain of CaMKII with binding score of −8.0 Kcal/mol (Fig. 12).

**Figure 13:**
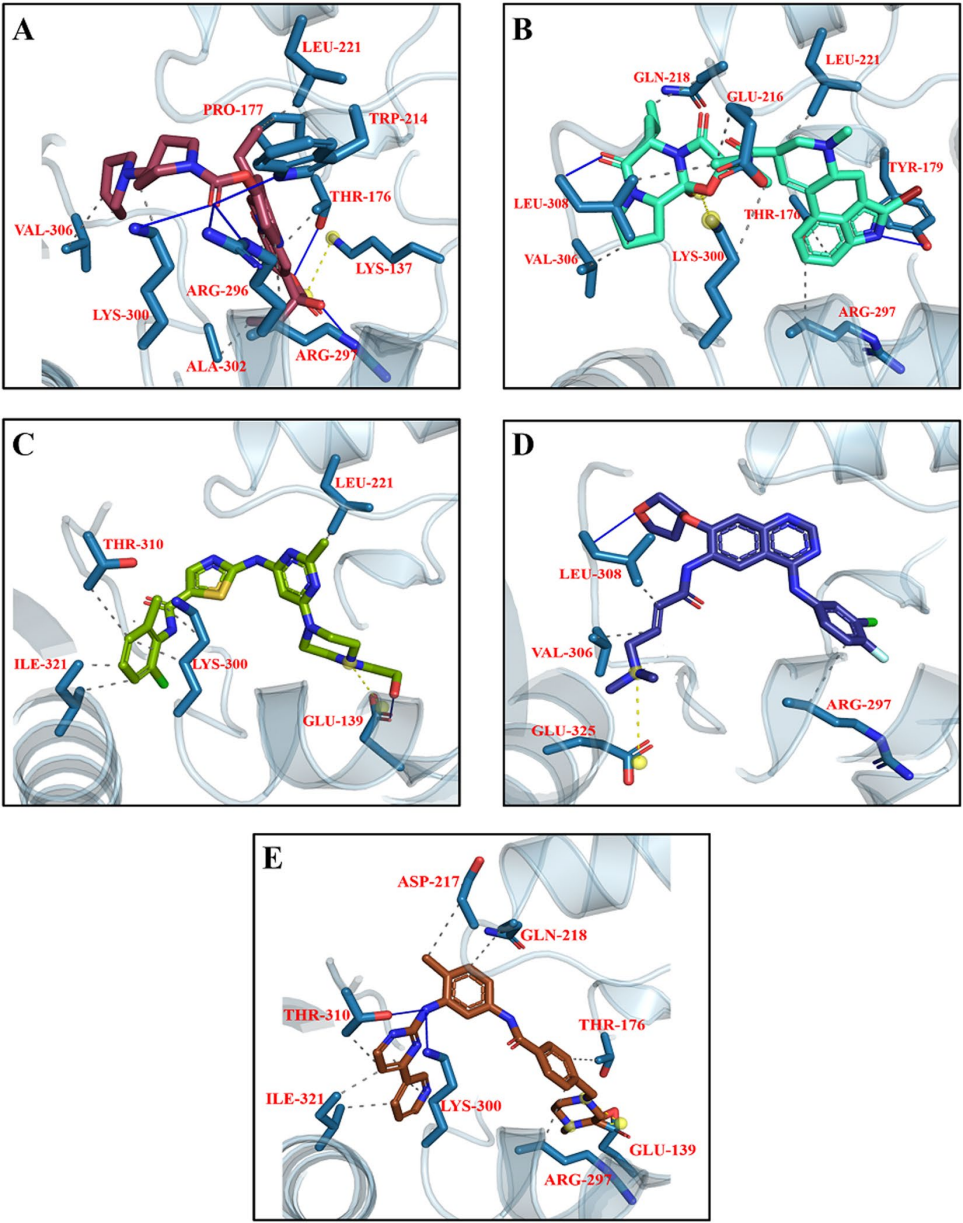
Top five docking conformations of CaMKII with (**A**) Irinotecan (maroon); (**B**) Bromocriptine (cyan); (**C**) Dasatinib (olive green); (**D**) Afatinib (blue); and (**E**) Imatinib (brown).

Library of compounds were docked into the active site of CaMKII with binding energies ranging from −10 to −4 Kcal/mol (Fig. S4). On the basis of binding affinities, our analysis suggested Irinotecan, Bromocriptine, Dasatinib, Afatinib, and Imatinib were having better affinity for CaMKII with scores of −10.2, −10.2, −9.6, −9.3, and −9.2 Kacl/mol, respectively, compared to Bosutinib. Irinotecan and Bromocriptine are having the same docking scores but bromocriptine having the highest binding affinity for CaMKII compared to Irinotecan. Dasatinib, Imatinib and Afatinib are also having the binding affinities comparable to docking scores (Table 5). All the five compounds showing interactions in the CaM binding domain where Lys 300, and Leu 308 involved in hydrophobic interactions while Arg 297 involved in H-bonding. Leu 221 in the kinase domain also showing hydrophobic and H-bond interactions with the compounds (Fig. 13; Table 5).

**Table 5:** Interacting residues of CaMKII with Irinotecan, Bromocriptine, Dasatinib, Afatinib, and Imatinib.

Drugs	Binding Affinity (−log_10_(K_D_|K_i_))	Protein Residue	Distance (Å)	Type of Interactions
Irinotecan	39.6	THR 176	3.21	Hydrophobic
		PRO 177	3.63	Hydrophobic
		LEU 221	2.33	Hydrophobic
		ARG 296	3.91	Hydrophobic
		LYS 300	3.70	Hydrophobic
		ALA 302	2.66	Hydrophobic
		LEU 308	2.93	Hydrophobic
		THR 176	2.73	H-bond
		TRP 214	2.10	H-bond
		ARG 296	1.78	H-bond
		ARG 297	2.04	H-bond
		LYS 300	2.74	H-bond
		LYS 137	4.48	Salt Bridge
Bromocriptine	42.8	THR 176	3.65	Hydrophobic
		GLU 216	3.19	Hydrophobic
		GLN 218	2.81	Hydrophobic
		LEU 221	2.79	Hydrophobic
		ARG 297	3.80	Hydrophobic
		LYS 300	3.76	Hydrophobic
		VAL 306	3.41	Hydrophobic
		LEU 308	2.37	Hydrophobic
		TYR 179	2.14	H-bond
		LEU 308	1.04	H-bond
		LYS 300	5.35	Salt bridge
Dasatinib	39.5	LEU 221	2.91	Hydrophobic
		LYS 300	3.78	Hydrophobic
		THR 310	3.88	Hydrophobic
		ILE 321	2.27	Hydrophobic
		GLU 139	3.53	H-bond
		GLU 139	4.82	Salt bridge
Afatinib	38.3	ARG 297	3.83	Hydrophobic
		VAL 306	3.73	Hydrophobic
		LEU 308	2.38	Hydrophobic
		LYS 300	1.36	H-bond
		Leu 308	2.53	H-bond
		GLU 325	5.03	Salt bridge
Imatinib	40.3	THR 176	3.82	Hydrophobic
		ASP 217	3.94	Hydrophobic
		GLN 218	3.91	Hydrophobic
		ARG 297	3.74	Hydrophobic
		LYS 300	3.41	Hydrophobic
		LEU 308	3.23	Hydrophobic
		THR 310	3.71	Hydrophobic
		ILE 321	3.06	Hydrophobic
		LYS 300	2.70	H-bond
		THR 310	2.77	H-bond
		GLU 139	3.34	Salt bridge

#### Interaction of Drugs with ERK

**Figure 14:**
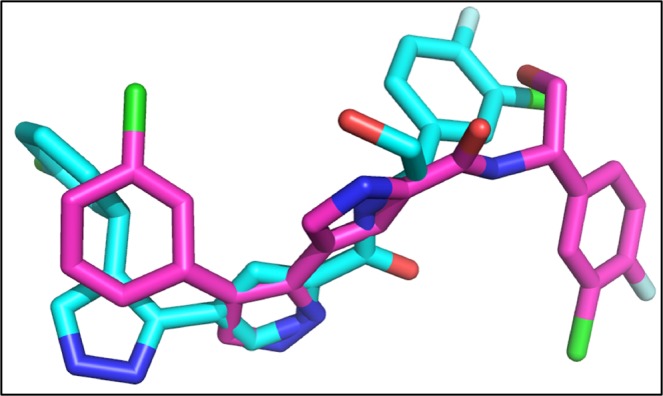
The co-crystallize and re-docked conformation of 82A. The co-crystallized ligand is shown in cyan while the re-docked ligand is shown in purple.

The re-docked conformation of 82A and the original co-crystallize conformation is shown in Fig. 14. The 82A bound to ERK with energy value of −8.4 Kcal/mol and RMSD of 3.3 Å.

**Figure 15:**
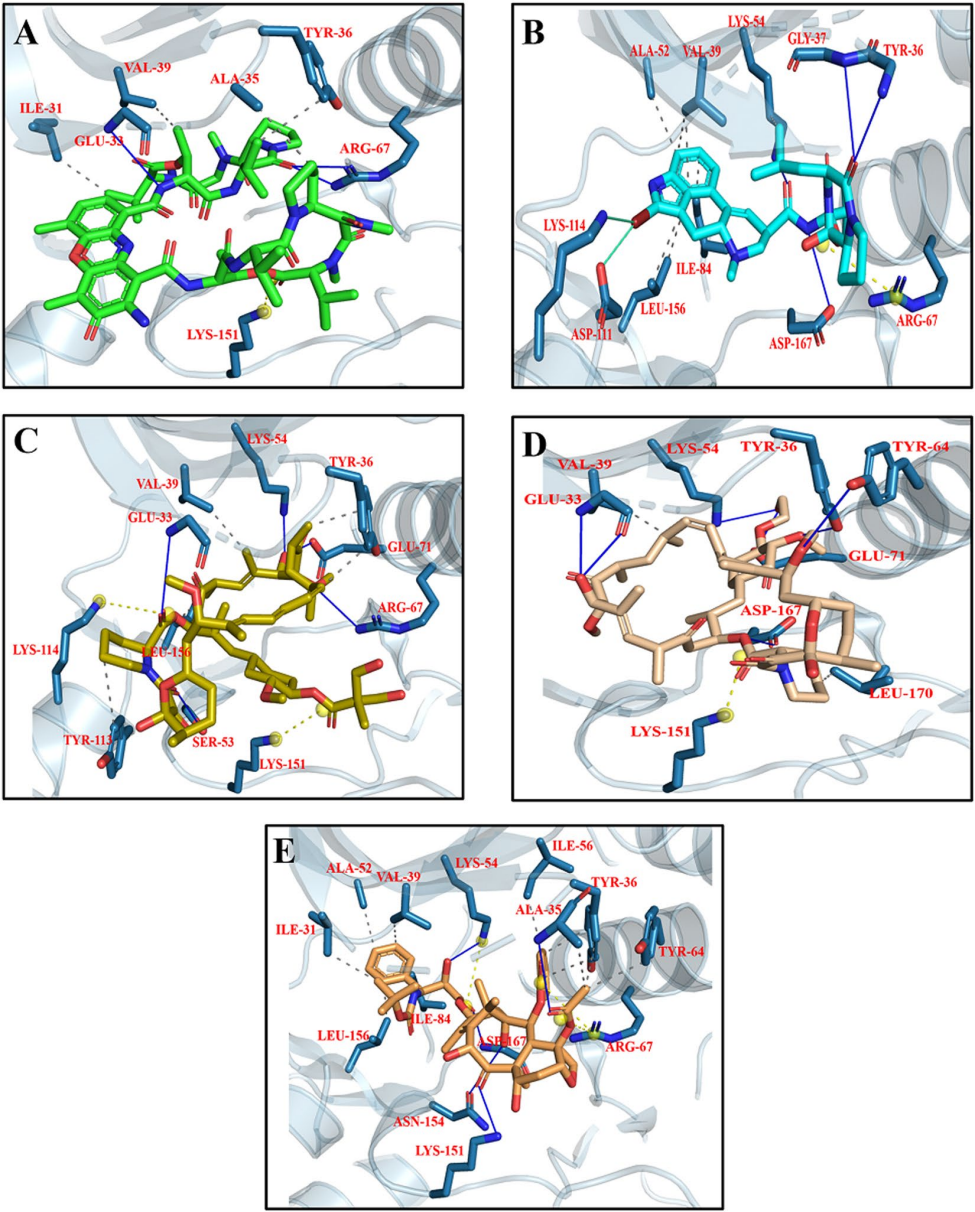
Top five docking conformations of ERK with (**A**) Dactinomycin (green); (**B**) Bromocriptine (cyan); (**C**) Temsirolimus (yellow); (**D**) Everolimus (beige); and (**E**) Docetaxel (golden).

The library of compounds were docked into the binding pocket of ERK and the detail scores of all the compounds is shown in Fig. S5. The results showed that the studied drugs are showing better affinity to ERK compared to original co-crystallize ligand. Dactinomycin, Bromocriptine, Temsirolimus, Everolimus, and docetaxel having −11.0, −10.3, −10.2, −10.1, −10.0 Kcal/mol binding affinity for ERK. On the basis of ligand binding affinities Everolimus having the highest affinity for ERK. Dactinomycin and Temsirolimus are having the same binding affinity value of 37 for ERK (Table 6). The interactions of ERK with the drugs showed that Tyr 36, Val 39 involved in hydrophobic while Lys 151 involved in H-bonding with all the five drugs (Fig. 15; Table 6).

**Table 6:** Interacting residues of ERK with Dactinomycin, Bromocriptine, Temsirolimus, Everolimus, and Docetaxel.

Drugs	Binding Affinity (−log_10_(K_D_|K_i_))	Protein Residue	Distance (Å)	Type of Interactions
Dactinomycin	37.7	ILE 31	3.63	Hydrophobic
		ALA 35	3.61	Hydrophobic
		TYR 36	3.96	Hydrophobic
		VAL 39	3.53	Hydrophobic
		GLU 53	3.49	H-bond
		ARG 67	2.18	H-bond
		LYS 151	4.00	Salt bridge
Bromocriptine	32.7	VAL 39	3.47	Hydrophobic
		ALA 52	3.71	Hydrophobic
		LYS 54	3.88	Hydrophobic
		ILE 84	3.06	Hydrophobic
		LEU 156	3.97	Hydrophobic
		TYR 36	2.08	H-bond
		GLY 37	3.45	H-bond
		LYS 54	2.17	H-bond
		ASP 167	2.74	H-bond
		ASP 111	3.15	Halogen bond
		LYS 114	3.92	Halogen bond
		ARG 67	4.55	Salt bridge
Temsirolimus	37.8	TYR 36	3.51	Hydrophobic
		VAL 39	3.34	Hydrophobic
		TYR 113	3.97	Hydrophobic
		LEU 156	3.70	Hydrophobic
		GLU 33	3.07	H-bond
		LYS 54	2.42	H-bond
		ARG 67	3.11	H-bond
		GLU 71	2.55	H-bond
		SER 153	2.98	H-bond
		LYS 114	5.29	Salt bridge
		LYS 151	3.93	Salt bridge
Everolimus	41.2	VAL 39	3.49	Hydrophobic
		LEU 170	3.72	Hydrophobic
		GLU 33	2.89	H-bond
		TYR 36	1.83	H-bond
		LYS 54	2.68	H-bond
		TYR 64	3.08	H-bond
		GLU 71	1.90	H-bond
		ASP 167	3.31	H-bond
		LYS 151	3.21	Salt bridge
Docetaxel	38.3	ILE 31	3.54	Hydrophobic
		ALA 35	3.76	Hydrophobic
		TYR 36	3.83	Hydrophobic
		VAL 39	3.50	Hydrophobic
		ALA 52	3.59	Hydrophobic
		ILE 56	3.49	Hydrophobic
		TYR 64	3.67	Hydrophobic
		ILE 84	3.26	Hydrophobic
		LEU 156	3.49	Hydrophobic
		ALA 35	3.09	H-bond
		LYS 54	2.73	H-bond
		LYS 151	2.21	H-bond
		ASN 154	3.42	H-bond
		ASP 167	2.57	H-bond
		LYS 54	5.04	Salt bridge
		ARG 67	4.76	Salt bridge

#### Interaction of Drugs with CBP

**Figure 16:**
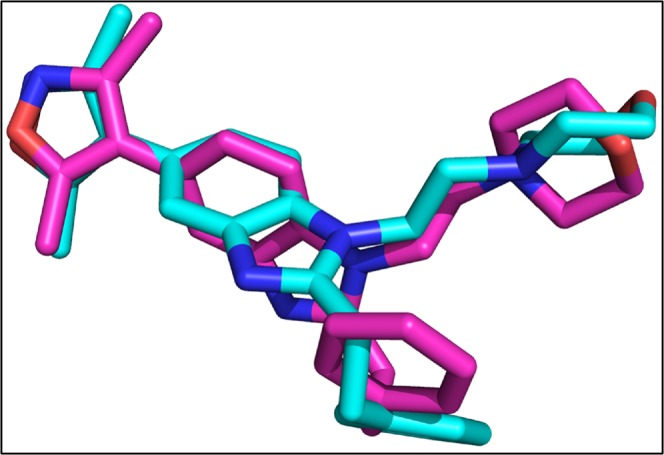
The co-crystallize and re-docked conformation of 2LL. The co-crystallize ligand is shown in cyan while re-docked ligand is shown in purple.

In case of CBP the original docked ligand is 2LL which was re-docked with binding score of −6.0 Kcal/mol and RMSD of 3.1 Å (Fig. 16). The docking of library of compounds were performed and the scores of each docking conformation is shown in Fig. S6. On the basis of scores, it is obvious that CBP does not showing high affinity for the studied drugs when compared with NMDA, AMPA, ERK, PKA, and CaMKII.

### Electrostatic potential of proteins

**Figure 17:**
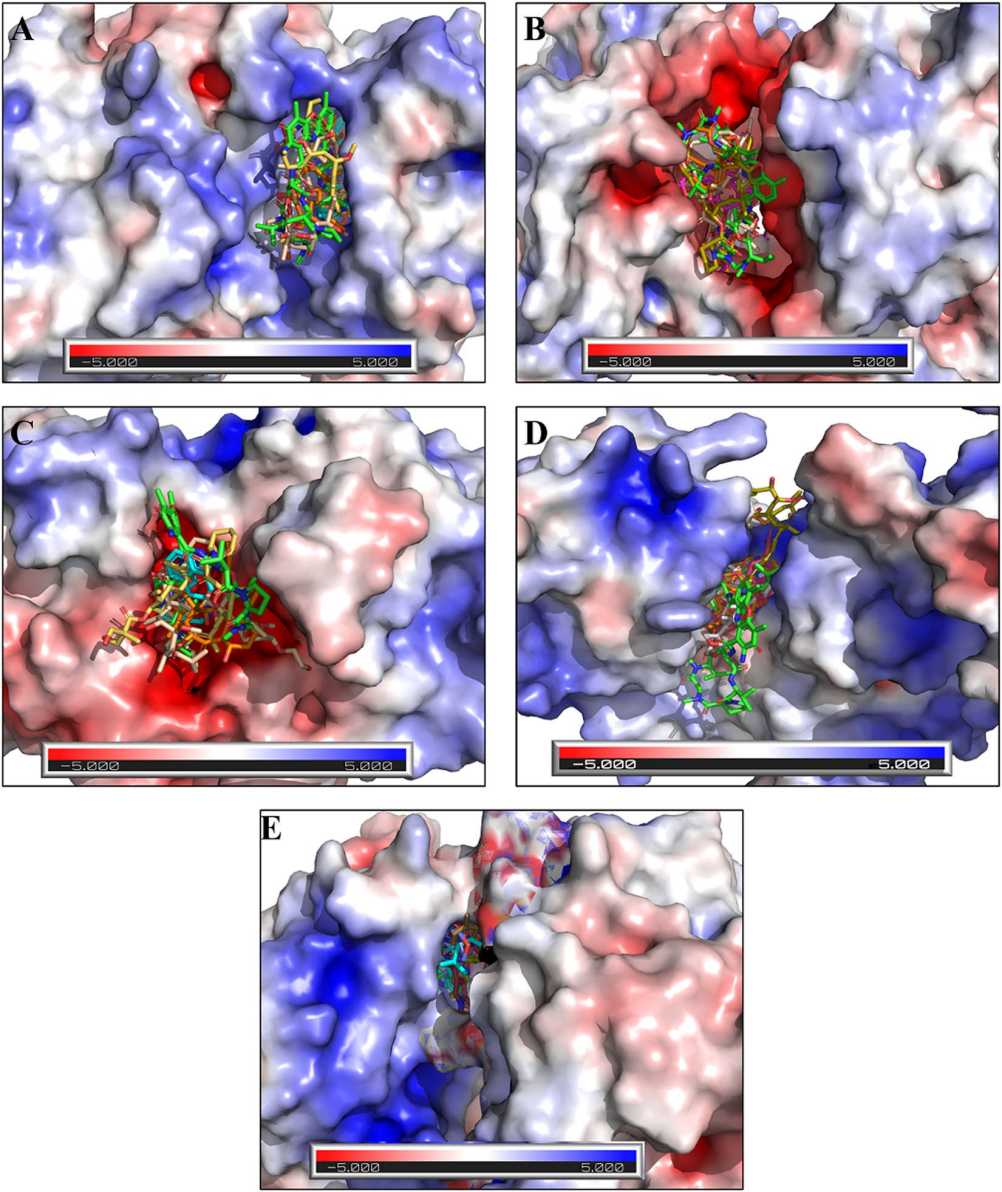
Electrostatic charge distribution at binding interfaces. (**A**) Electrostatic potential map of ERK complexed with Dactinomycin, Bromoriptine, Temsirolimus, Everolimus and Docetaxel. (**B**) Electrostatic potential map of NMDA complexed with Dactinomycin, Temsirolimus, Everolimus Docetaxel and Teniposide; (**C**) Electrostatic potential map of PKA complexed with Dactinomycin, Temsirolimus, Everolimus Docetaxel and Bromocriptine; (**D**) Electrostatic potential map of AMPA complexed with Dactinomycin, Temsirolimus, Paclitaxel, Vincristine and Irinotecan; (**E**) Electrostatic potential map of CaMKII complexed with Irinotecan, Bromocriptine, Dasatinib, Afatinib and Imatinib.

The electrostatic potential of NMDA, AMPA, ERK, PKA, and CaMKII is shown in Fig. 17. On the basis of electrostatic potential the active site of PKA, and NMDA is more electronegative, in case of ERK it is more electronegative, in case of AMPA it is having the electropositive as well as electronegative residues while in case of CaMKII it is almost neutral. The electropositivity and electronegativity favors the strong interactions of ligand with the proteins which is also evident with the binding affinity of Dactinomycin, Temsirolimus, and Everolimus. These drugs are showing highest binding affinity for NMDA, ERK, PKA, and AMPA. While in the neutral binding pocket of CaMKII, Irrinoteacn and Bromocriptine having the high binding affinity.

### Plif analysis

The protein ligand interaction fingerprints were calculated using MOE. Dactinomycin, Temsirolimus and Everolimus are the drugs that are showing maximum affinity for NMDA, AMPA, PKA and ERK, hence the PLIF analysis were performed for all the three drugs in order to find any common functional group in all the compounds. On the basis of PLIF analysis it is revealed that due to large molecular structure of Dactinomycin, it has many interacting points with the active site residues of NMDA, AMPA, PKA, and ERK. It can involve in H-bonding due to the presence of carboxyl group as well as in hydrophobic interactions due to the presence of benzene ring (Fig. S7). Temsirolimus can also involve in H-bonding due to the presence of terminal hydroxyl group and also in hydrophobic interactions with the help of long alkane chain with the active site residues of NMDA, PKA, AMPA, and ERK (Fig. S8). Everolimus is involve in H-bonding with the help of its hydroxyl and carboxyl group while its pyrimidine ring involved in hydrophobic interactions with NMDA, PKA and ERK (Fig. S9).

## Discussion

The neurons of central and peripheral nervous system as well as oligodendrocytes are particularly susceptible to off target side effects pitched in by chemotherapy^30,84^. These off target effects may contemplate into revamping functions of both dividing and non-dividing cells both at central and peripheral levels. The proposed mechanisms put forwarded to explain these pathologies include faulty DNA repair mechanisms, blood brain barrier dysfunction, disordered immune regulation and impaired neurotransmitter signaling^85^. There is an increasing evidence that CICI disrupts neurogenesis particularly in adult hippocampus^14,16^. The implication of such reformation results in alteration of hippocampal neural circuitry which critically affects not only memory formation and learning acquisition, but also interregional articulation of anatomically distant but functionally cognate brain regions^86^. This can be further inferred from the reported clinical evidence of frontal cortical deficits by chemobrain^87^. Therefore, synaptic plasticity functions mediated by neurons of hippocampus are at stake.

The focus of the study is to investigate the interactions of various chemotherapeutic drugs against major proteins involved in LTP pathway, by docking algorithm as their interacting residues may provide useful insight into functional alteration which can be related to cognitive processes. The results derived from this study revealed that Dactinomycin, Temsirolimus, Everolimus, Docetaxel and Teniposide are top 5 drugs interacting with NMDA residues located in Ligand Binding Domain (LBD) of GluN2 near Glutamate binding pocket (Fig. 7). Contextually, NMDARs have been focus of pharmacological modulation by virtue of allosteric modulators, however, limited by excessive off-target effects in lieu of excessive NMDAR inhibition. Notably, the competitive antagonists of two main substrates of NMDARs i.e. Glycine and Glutamate, targets LBD of GluN1 AND GluN2 respectively, while the Transmembrane Domain (TMD) is targeted by the channel blockers^88^. Interestingly, Dactinomycin has been reported to rescue retinal ganglion cells from NMDA mediated excitotoxicity, suggesting potential evidence of Dactinomycin in partial inhibition of NMDA receptor^89^. The mTOR inhibitor Everolimus has been tested in Phase 1 clinical trials for targeting glutamatergic signaling for Autism Spectrum Disorders (ASDs) and Temsirolimus have similarly been reported to be used in rodents for altering mTORpathway^90–92^.

Under physiological conditions, the simultaneous closure of GluN1/N2 aided by the binding energies in the advent of agonist (Glycine & Glutamate) binding puts the TMD to undergo conformational changes to open the channel^93,94^. However, binding of NMDA-R competitive antagonist arrests the NMDA gating mechanism to an open cleft conformation of GluN1/N2 thus blocking the channel. This suggest that these drugs while interacting with residues on GluN1/N2 can interfere with NMDA gating mechanism. Specifically increased intracellular Ca^+2^ influx as a result of NMDA activation leading to increased intracellular Na^+^ by virtue of AMPA over activation may causes swelling of neuronal cell body^95^. However, it is difficult to deduce whether interactions by compounds mimicking competitive antagonists can induce allosteric modulation on the positive or negative side. The residues Tyr 754 and Ile755 have reported to influence the binding affinities of NMDA competitive antagonist. Moreover, the NAM NVP-AAM077 and ST3 binds cavity in GluN2A harboring non conserved residues Tyr 754 and Ile755. Interestingly, these are among the same residues which have shown interaction with Dactinomycin, Temsirolimus, Everolimus, Docetaxel and Teniposide in our study. The residue Tyr754 had been proven to be of detrimental importance in site directed mutagenesis replacement with Lysine^96^. This replacement boosted the glutamate potency thus re-sculpting the conformational states of GluN2A and altering the general behavior of glutamate binding site in GluN2A. Perplexingly, the same residue along with Lys738, also effect the Glycine binding site on GluN1. The neighboring residue Ile755 have also reported to modulate the binding cavity for negative NMDA allosteric modulators (NAMs)^96^. The functional implication of these findings favors a potential competitive antagonistic action with possible partial NMDA inhibition. Interestingly, peripheral NMDA receptors are also drug targets to evade multidrug resistance in cancer by virtue of their ability to downregulate ABC transporters^97^. Such is case of compound MK-801, which noncompetitively antagonizes NMDA receptor^98^.

The AMPARs are another family members of iGluRs with structurally similar homo/hetero tetrameric organization from GluA1-A4, however differing in its preferentially Na^+^ permeability, resulting in rapid postsynaptic depolarization^99,100^. The AMPARs have shown interactions with Dactinomycin, Temsirolimus, Paclitaxel, Vincristine, and Irinotecan with lowest binding energies among the current study’s selected drug dataset (Fig. 9). Related to our findings, the role of mTORinhibitor Temsirolimus in targeting AMPAR have been reported before^90^. To add further, Taxol has also been previously reported to selectively repress cationic influx in glutamate excitotoxicity^101^. Paclitaxel and Vincristine have been previously documented for their involvement in mediating chemotherapy induced peripheral neuropathy (CIPN), having toxic effect on dorsal root ganglion neurons^1,102^. However the CIPN damaging effect is also partly mediated by the induction of strong inflammatory component^103^. Similarly, Irinotecan have been documented previously for their reduction in excitatory neurotransmission by interfering with pre and post synaptic gene expression^104,105^. Moreover, clinical deterioration in cognitive decline in patients receiving irinotecan in combination with 5’Flourouracil has also been reported^106^.

AMPARs have been tried with therapeutic inhibition in case of epilepsy^107^. Past studies also suggest that triggering multiple topological conformation in the face of activity dependent interactions in AMPARs alter its mobility which may leads to its desensitization^108^.

The current study findings showed off target interaction of Temsirolimus, Vincristine and Irinotecan with Glu 402 residue and Thr686 of GluA2 subunit of AMPA. Previous crystal structure studies of AMPAR reported interaction of Glu402 residue of subunit with Thr 686 residue which are positioned at corners of binding cleft and contributes to the stability of closed conformational state^109^. Mutations at either of these residues result in fall of agonist binding affinities and efficacy^110–113^. Moreover, Hogner *et al*. reported that the inter-domain steric hindrances or remodeling of Glu402-Thr686 interaction can readjust the course of domain closure, independent of ligand affinity^109^. Interestingly, the AMPA antagonist DNQX targets the same Glu402- Thr 686 interaction^114^. The Gly731 residue participate in cleft closure from open to closed state^115^. The close receptor conformational state has been associated with full agonistic behavior while a partial agonist receptor renders incomplete receptor closure. Interestingly, the L605T mutants of LBD of iGluR2 showed contrary results.

All five top docking complexes exhibit interaction with residue Tyr450. Interestingly substitution of Tyr with Ala at this position has resulted in diminution of glutamate potency^116^. Similarly, off target interactions have also been observed at position Glu705, which had been reported to eliminate agonist binding on experimental mutation. Furthermore, experimental mutation by Armstrong *et al*., for residue Leu650 to Thr decrease the potency of AMPA receptors to Glutamate by 8.5 folds^117^.

The GluA2 subunit of AMPA also harbors interacting residues Ser654, Thr655 and Phe658, which are flexible meta-interaction site and mutation in this region may alter binding kinetics culminating into aberrant AMPA receptor activation^118^. Residues Tyr450, Pro478,Thr480, Ser654 AND Glu705, all directly interact with ligand to mediate strong binding^115^. The GluA2 subunit critically regulates biophysical function by keeping in check the receptor kinetics and Ca^+2^ permeability. It is noteworthy that slowing down the AMPAR deactivation and sensitization by the use of allosteric modulators as Diazoxide^119^ and Aniracetam^120^ is tried in clinical studies for cognitive impairment and depression. Puzzlingly, slowing or blocking desensitization in transgenic animals led to lethal outcomes^121^. The cumulative endpoint from past studies related with our off target interaction suggested that the deactivation kinetics of AMPARs is highly variable and any untoward off-target interaction particularly within the regions of ligand binding can potentially enhance or delay AMPA deactivation thus dysregulating excitatory postsynaptic potential (EPSP).

Evolutionarily, kinases are generally conserved and receive a high competition for substrate binding particularly from ATP in mM concentrations, thus decreasing their probability to be involved in off-target interactions^122^, however, with the development of tyrosine kinase inhibitors and their use particularly in cancer patients, off-target interactions are not very unlikely^123^. During LTP induction, the activity of NMDA leads to surge of intracellular Ca^+2^ triggering a biochemical cascade emanating at AMPAR mediated EPSC. This biochemical cascade is driven by CaMKII, an unusual kinase, capable of auto-phosphorylating itself (at residue T286 and T287), mediated by Ca^+2^ and Calmodulin^124^. Moreover, it is further capable of holding its activated, the ‘autonomous state’, even though the initial stimulation is hold off i.e. more specifically speaking, even when the intracellular Ca^+2^ vanishes thus behaving like a ‘molecular switch’^125^. With reference to top interacting drugs with CaMKII, the list include Irinotecan, Bromocriptine, Dasatinib, Afatinib, and Imatinib (Fig. 13). Irinotecan has been previously reported to enhance neurite outgrowth by structural similarity of Polysialic Acid^126^. Recently, the tyrosine kinase inhibitors have been shown to increase oxidative stress with resultant activation of CaMKII in cardiac fibroblasts^127^. Interestingly, Imatinib, the first approved kinase inhibitor had been known for remarkable selectivity^128–130^ yet in our study it is one of the top contenders for off target interaction with CaMKII. The kinase inhibitors are however speculated to inhibit 10–100 kinases suggesting low selectivity^130,131^.

Structurally, CaMKII have 12 subunits, each having a carboxy terminal, the Hub region, followed by a regulatory segment which harbors PTM segments for phosphorylation, NAc-Glycosylation, oxidation at position Thr287, Ser280 and Met 281 & 282 respectively^76,132,133^. The present work analysis suggests that chemotherapeutic drugs are exhibiting off targeting interactions in the regulatory segment which spans between the residues 273–317 and the kinase domain. Residues in this region and particularly the residue Thr 286 has been proven to be essential as knocking it down will abolish LTP induction with significant memory deficits^134,135^. The important residues participating in off target interactions include Arg 296, Arg 297, Met307 which falls in regulatory segment more specifically in the CaM recognition sequence (residues 290–314) while the residue Glu 216 comes under kinase domain. The residue Arg 297 lies at the interface of regulatory and kinase domains and is involved in hydrophobic interactions with kinase domains of other subunits. Any interference in the CaM recognition sequence may results in alteration of Ca + 2 trapping which is very crucial for the autonomous phosphorylation activity of CaMKII. Moreover, another off-target interacting residue Met 307 is in very close proximity to the Calmodulin footprint which is on residue Thr 305 and 306 and phosphorylation of these residues will abrogate CaM/Calmodulin binding^136^ and binding with α-actinin which aids in CaMKII anchoring with NMDA^137^.

Another cellular kinase the Protein Kinase A (PKA), contributes to LTP induction. PKA since their discovery by Kreb and coworkers, have been known to be important second messenger after cAMP^138^. The regulatory subunit spans on multiple domains and harbors binding site for cAMP, dimerization-docking domain and a linker segment, while the catalytic subunit harbors two phosphorylation sites, one at residue Ser 338 which is near the C-terminal and the other is on residue Thr 197, coming under the activation loop/segment that serve as the docking site for protein substrate^139^. Recent literature suggest appearance of memory deficits and long term depression (LTD) on deletion of regulatory subunits^140^. The current investigative analysis reveals major off-target interaction from Dactinomycin, Temsirolimus, Everolimus, Docetaxel and Bromocriptine (Fig. 11). In this frame of reference, Dactinomycin has been previously reported to inhibit kinase activity by interfering at the SH2 domain which is crucial for ligand induced activation^141^. Reduction of intracellular signal transduction by modulation of protein kinases have been reported with Bromocriptine^142^. Contrarily, taxanes have been previously reported to pathologically enhance kinase activity promoting cell survival^143,144^.

The off-target interactions have been shown to be involving the residues Gly 50, Thr 51, Gly 52, Ser 53, which are part of the Glycine rich loop (spanning from residue 50–55), an integral part of ATP binding site, and the residue Glu 170, part of ribose pocket, which carries the phosphate binding cassette^145^. Interestingly, the Glycine rich loop is one of the targeted sites for the development of PKA inhibitors^77^. Moreover, off-target interactions have also been observed in the activation loop hooking residues Phe 187, Thr 201, Pro 202 and Glu 203. Notably, the activation loop when dephosphorylated, retains in intrinsically disordered conformation^139^. The residues from the catalytic loop have also been involved in off-target interaction.

Two decades back, English and Sweatt reported for the first time, direct involvement of extracellular signal regulated kinase (ERK) in synaptic plasticity and later it was also established that ERK activation is detrimental for L-LTP^146,147^. This observation was further supplemented by the works of Gooney *et al*. who suggested requirement of ERK activation for BDNF induced LTP^148^. ERK Kinase 1 and 2 which are important for LTP pathway, are related to mitogen activated protein kinase (MAPK) superfamily^149^. Both human ERK1 and ERK2 share 84% identity and activated on parallel levels when stimulated^150,151^.

The results suggest Dactinomycin, Bromocriptine, Temosirolimus, Everolimus and Docetaxel among top lowest scorers of mediating off target interactions with ERK2 protein (Fig. 15). All of these interactions have been appeared in protein kinase domain (23–313 residue). All these drugs exhibited interactions in the Glycine rich residues spanning between 32–37 residues. Dactinomycin is associating through hydrophobic interactions on Ala35 and Tyr36, Temisrolimus via Tyr36 hydrophobically and through Hydrogen bonding on residue Glu33. Hydrogen bonding is also on residue Glu33 and Tyr36 by Everolimus while Docetaxel is interacting on residue Ala35 both through H-bonding and hydrophobically while residue Tyr36 is receiving only hydrophobic interactions. The Lys54 residue which mediates coupling of ATP phosphates to α helix is target of off-target Hydrogen bonding by Bromocriptine, Temosirolimus, Everolimus and Docetaxel. Temsirolimus and Everolimus, both are engaged to Glu71 via Hydrogen bonding. Everolimus and Docetaxel are interfering in the corner of activation segment on the residue Asp167 by hydrogen bonding.

CBP are transcriptional coactivators known for gearing transcriptional expression of genes involved in cell survival. Moreover, CBP has also been linked to chromatin remodeling and in mediating acetyltransferase reactions^72^. CBP mutation in Rubinstein-Taybi Syndrome has shown to be critically detrimental in LTP pathway^152^. CREB and CBP are tools of cellular machinery imparting a central role in LTP and memory consolidation^153^. Specifically, CREB and CBP are positioned downstream in LTP signaling cascade and phosphorylation of Serine 133 on CREB results in its activation. This triggers CREB to interact with CBP^154,155^ via the same Ser 133 site, inducing gene expression.

The results suggest Temsirolimus, Docetaxel and Everolimus to be binding with lowest energies, in the Bromo Domain region (BRD) of CBP, however, the scores are around −8 k cal/mol. The scores are less than the top docking scores selected for studying interaction. Interestingly, the BRD has attracted particular target of interest for development of CBP BRD inhibitors^156^. Contextually, the CBP BRD inhibition has also been linked to modulate RGS4 expression, involved in GTPase activation, by the use of Temsirolimus^157^.

The molecular electrostatic potential analysis of the LTP pathway proteins (Fig. 17) reflected primarily electronegative charge dominance in case of NMDA and PKA while electropositivity was observed more in case of ERK and AMPA. CaMKII stands in a position where the binding interfaces is not depicting overall charge electropositivity or electronegativity which suggest its favor more towards hydrophobic interaction.

The development of kinase inhibitors have been increased tremendously during the last decade with more than 250 kinase inhibitors in the testable phase of clinical trials^158^ However, the selectivity of kinase inhibitors have been in question by invitro studies previously^159^. The current study findings are in alignment to the findings reported before, however, the current study identified potential offtarget interactions, suggesting new targets where the kinase inhibitors can be studied. Such is the case in mTOR signalling which has been associated with various neuropathlogies such as Autism, Epilepsy, Feeding behavior and age related synaptic alterations^160–163^. Moreover, mTOR pathway has been linked to LTP & Long term depression (LTD) pathways by virtue of synaptic protein translation^164,165^. Therefore, interaction of mTOR inhibitors such as Everolimus & Temsirolimus can be detrimental. Since the chemotherapy drugs are primarily developed with the intention of targeting cancer cell proteins, the proteins with similar features are present in body elsewhere. The interaction of chemotherapeutic drugs with neuronal proteins suggest an area which is difficult to study directly on human tissues.

Moreover, some chemotherapeutic drugs, the kinase inhibitors are in clinical evaluation for treating other neurological diseases^58^. Our study focused on some major proteins involved in LTP Pathway, however detailed elucidation of LTP dysregulation by chemotherapy requires investigation of all other proteins involved in LTP having other isoforms. Such is the case with Protein Kinase C zeta PKCζ^166^ which is also a very important protein in LTP pathway. The absence of crystal structures of PKCζ and the intrinsically disordered nature of CREB protein^167^ limited our interaction analysis.

The spatiotemporal kinetics of LTP proteins affected by chemotherapy can also provide useful insight. Furthermore, receptor proteins involved in LTP like NMDA and AMPA are appareled with multitude of regulatory sites imparting sensitive properties to afferent stimulus, therefore, our study’s result will be case of over simplification if we generalize our findings to all NMDAR or AMPAR functionally diversified subunits. The functional implication of the off targets corresponds to the importance of LTP pathway which is crucial in neuronal learning and memory processes. On a general note, although our study identified interactions which may be linked to aberrant LTP functioning, the mechanism of chemobrain is also well reported to be linked to cytokine dysregulation and other mechanism discussed above. Therefore, chemotherapy may be on an off-targeted route, be interacting with other pathways directly linked to the proposed mechanisms of causing chemobrain, and all of these etiologies can synergistically define the resultant disease outcome of cognitive decline. Having said that, explaining the intricacies of actual proteins and pathways involved in chemobrain by off target interactions is a relatively deserted area and our study in this context may add potential links to further explore not only the chemobrain mechanisms but also to understand the LTP affected by drug target interactions.

## Conclusions

The findings of this study suggest chemotherapy drugs to be interacting with LTP pathway proteins, which may modulate the induction and maintenance of E-LTP and L-LTP phases. As LTP is directly linked to synaptic mediation of learning acquisition and memory consolidation, the already reported aftermath of cognitive decline in chemobrain by altered LTP signaling is further objectively substantiated by this study. Moreover, regarding chemotherapy compounds in current clinical use, this study has provided novel aspects related to drug repurposing and predictive toxicology, which can help in development of more effective yet more tolerable chemotherapeutic drugs. However, further studies will be required to illustrate the agnostic and antagonistic effects of chemotherapy on LTP pathway.

## Supplementary information

Supplementary Material
