# Supplementary material for: The Route to ‘Chemobrain’ - Computational probing of neuronal LTP pathway
Source: Sci Rep. 2019 Jul 3;9:9630. doi: 10.1038/s41598-019-45883-9 (PMC6610097; doi:10.1038/s41598-019-45883-9)
Supplement: Supplementary file 1 — Supplementary Material [file 41598_2019_45883_MOESM1_ESM.pdf]

The Route to 'Chemobrain' - Computational probing of neuronal LTP pathway

Ammad Fahim[1]*, Zaira Rehman[1], Muhammad Faraz Bhatti[1]*, NasarVirk[1], Amjad Ali[1], Amir Rashid[3], Rehan Zafar Paracha[2]*

1 Atta ur Rahman School of Applied Biosciences, National University of Sciences and Technology (NUST), Islamabad, Pakistan

2 Research Centre for Modeling and Simulation, National University of Sciences and Technology (NUST), Islamabad, Pakistan

3 Department of Biochemistry, National University of Medical Sciences, Rawalpindi, Pakistan

Authors to whom correspondence should be addressed:
Ammad Fahim
[1*]Atta ur Rahman School of Applied Biosciences (ASAB), National University of Sciences and Technology (NUST), Sector H-12, Islamabad 44000, Pakistan, afahim.phdabs04asab@asab.nust.edu.pk
Muhammad Faraz Bhatti[1*] Tel. Office: +92 51 9085 6136, Fax # +92 51 9085 6102
[1*]Atta ur Rahman School of Applied Biosciences (ASAB), National University of Sciences and Technology (NUST), Sector H-12, Islamabad 44000, Pakistan, mfbhatti@asab.nust.edu.pk
Rehan Zafar Paracha[2*]
[2*]Research Centre for Modeling and Simulation (RCMS), National University of Sciences and Technology (NUST), Sector H-12, Islamabad 44000, Pakistan, rehan@rcms.nust.edu.pk

Supplementary Material:

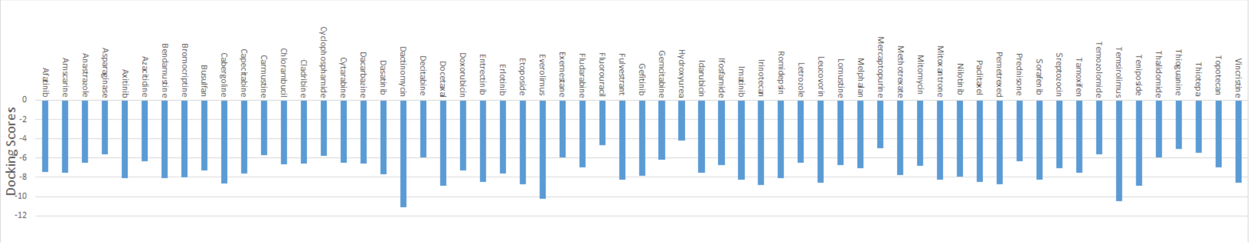

Figure S1: The docking scores of all the ligand-protein complexes of NMDA. X-axis represent the name of drugs while Y-axis represent the docking scores.

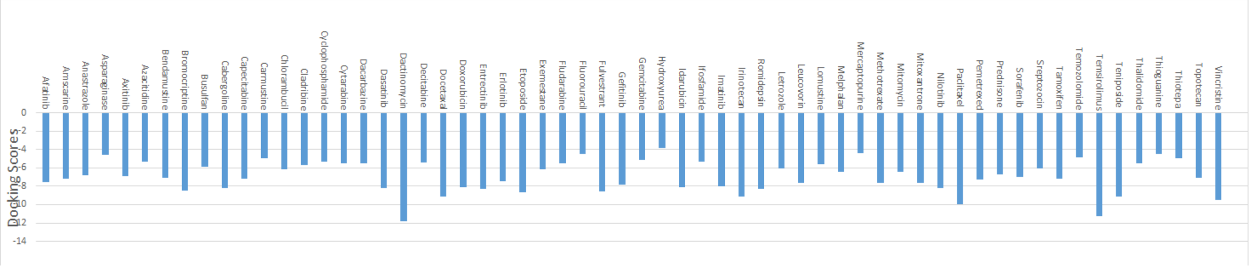

Figure S2: The docking scores of all the ligand-protein complexes of AMPA. X-axis represent the name of drugs while Y-axis represent the docking scores.

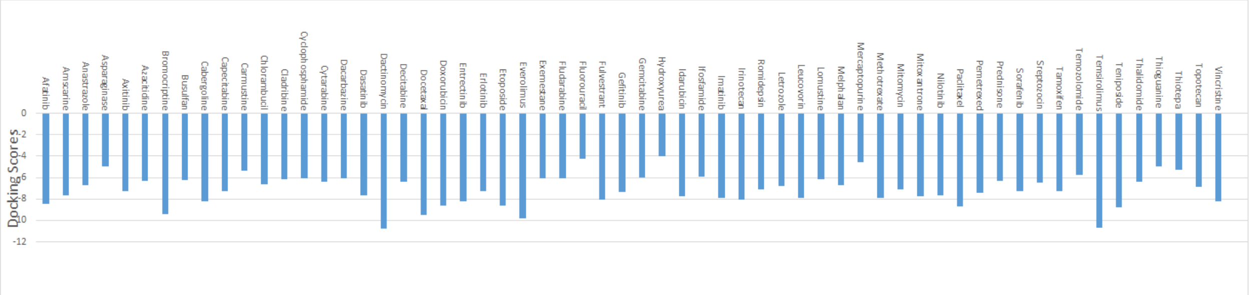

Figure S3: The docking scores of all the ligand-protein complexes of PKA. X-axis represent the name of drugs while Y-axis represent the docking scores.

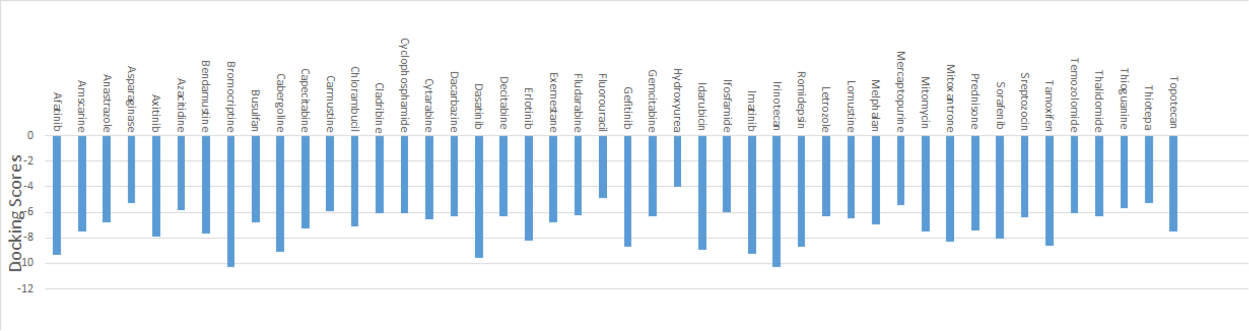

Figure S4: The docking scores of all the ligand-protein complexes of CaMKII. X-axis represent the name of drugs while Y-axis represent the docking scores.

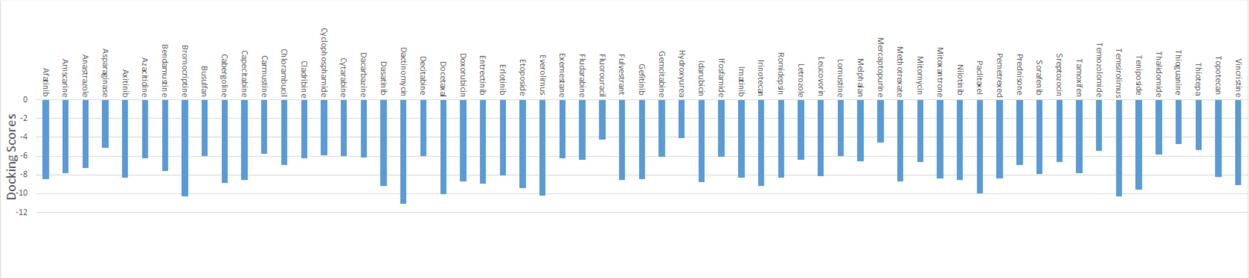

Figure S5: The docking scores of all the ligand-protein complexes of ERK. X-axis represent the name of drugs while Y-axis represent the docking scores.

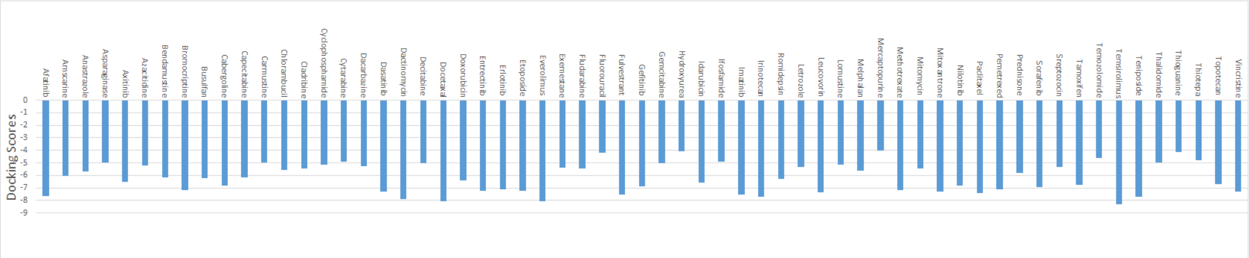

Figure S6: The docking scores of all the ligand-protein complexes of CBP. X-axis represent the name of drugs while Y-axis represent the docking scores.

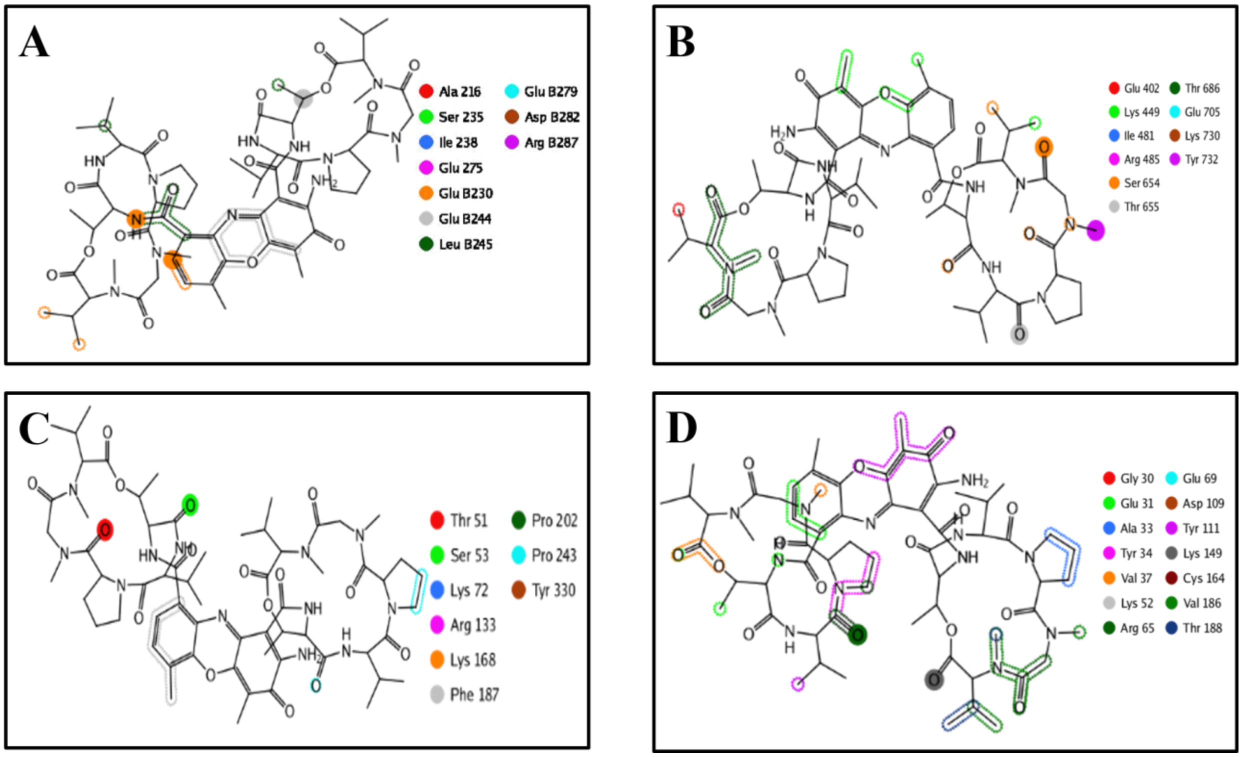

Figure S7: Protein ligand interaction fingerprints of Dactinomycin docked with (A) NMDA; (B) AMPA; (C) PKA; (D)ERK.

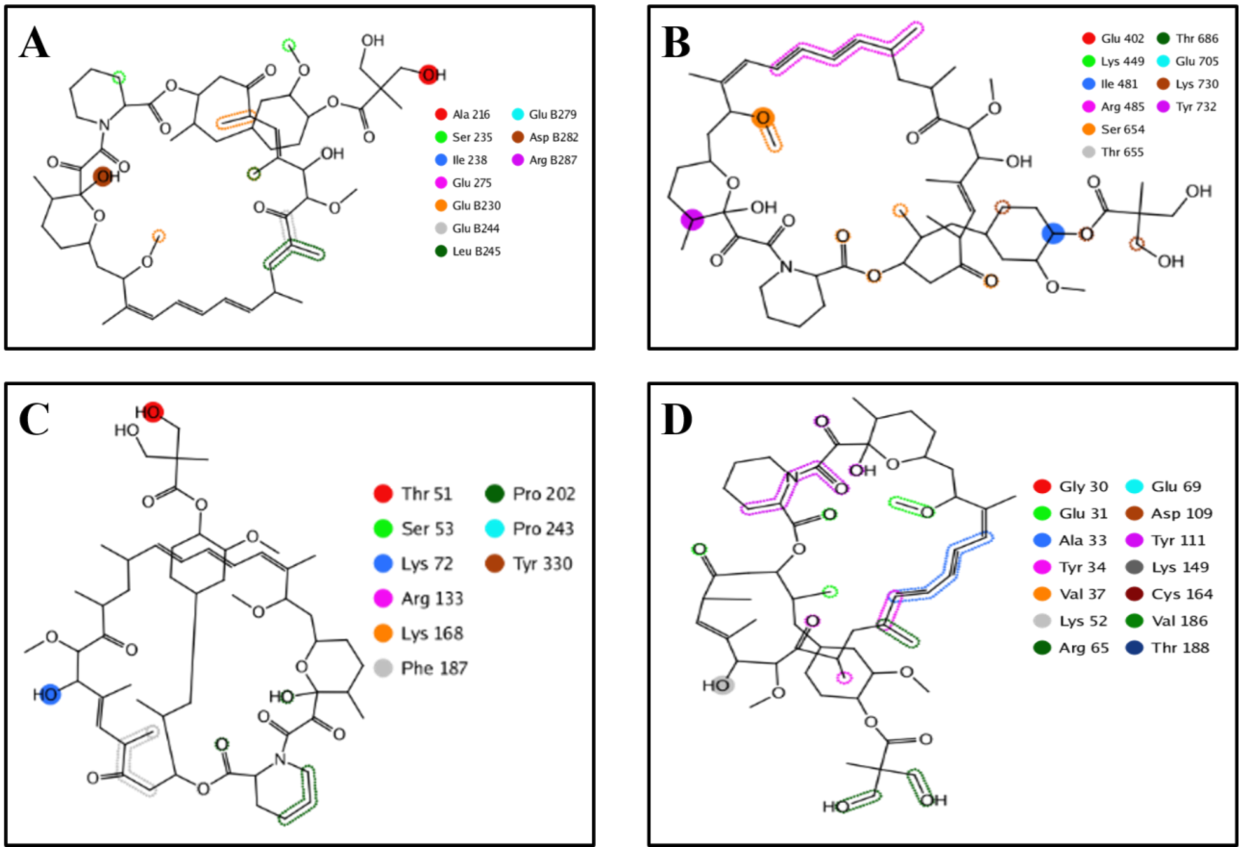

Figure S8: Protein ligand interaction fingerprints of Temsirolimus docked with (A) NMDA; (B) AMPA; (C) PKA; (D)ERK.

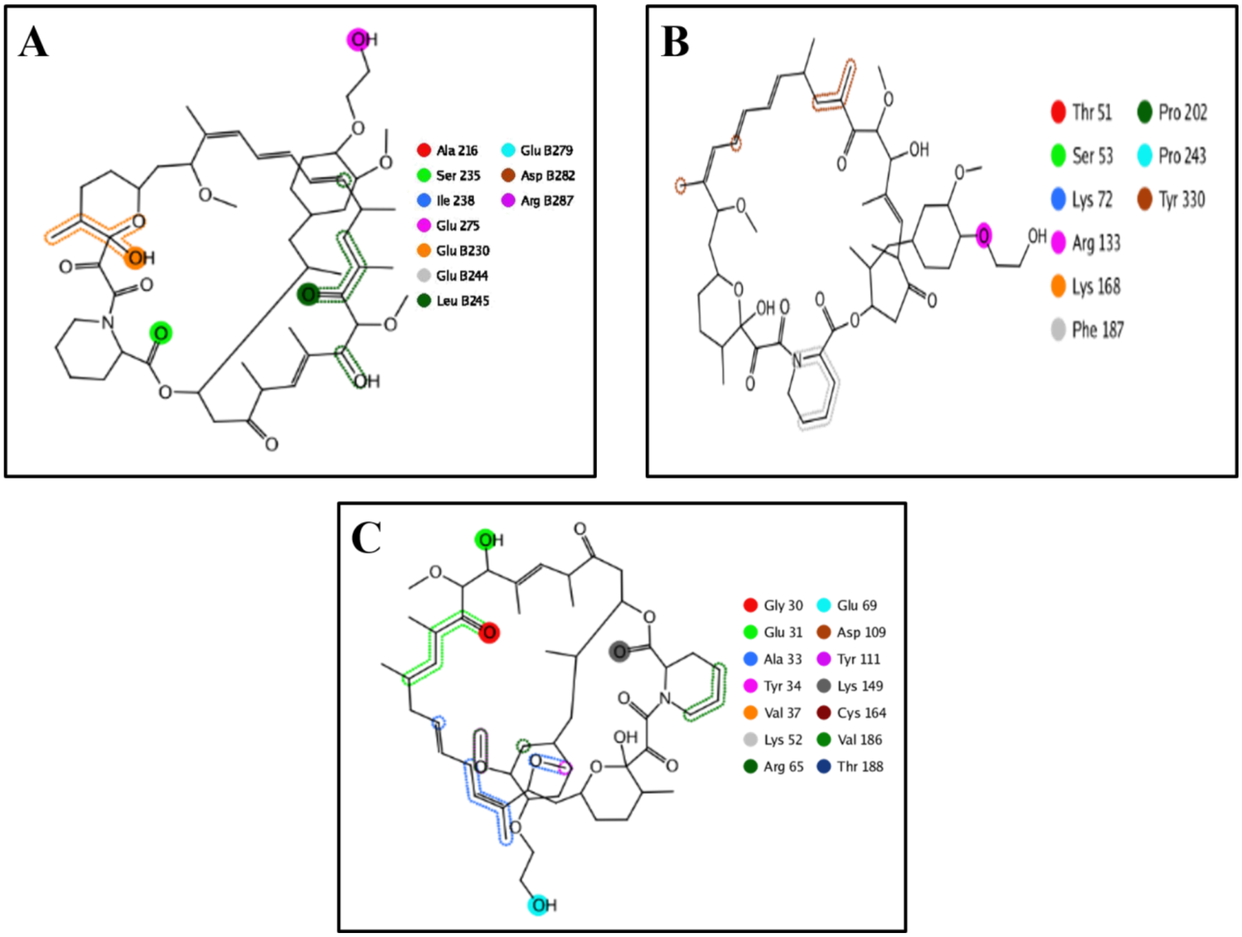

Figure S9: Protein ligand interaction fingerprints of Everolimus docked with (A) NMDA; (B) PKA (C) ERK.
